# S100A14 in Tumor‐Derived EVs Targets PIAS3 to Reprogram Astrocytes and Induce Immunosuppressive Microenvironment Promoting Brain Metastasis and Germacrone Reversal Effect

**DOI:** 10.1002/advs.202522921

**Published:** 2026-04-10

**Authors:** Qian Feng, Xia Yang, Lin An, Xue‐Yu Wang, Ming‐Rui Wang, Zhuo‐Hong Cai, Cai‐Jun Xie, Li‐Jun Qiao, You‐Bi Shen, Xi‐Min Liang, Rong Zhang, Cai‐Yan Wang, Zhong‐Qiu Liu, Rong‐Rong Zhang

**Affiliations:** ^1^ Guangdong Provincial Key Laboratory of Translational Chinese Medicine, Joint International Research Laboratory of Translational Cancer Research of Chinese Medicines International Institute for Translational Chinese Medicine School of Pharmaceutical Sciences Guangzhou University of Chinese Medicine Guangzhou China; ^2^ Department of Neurosurgery the Second Affiliated Hospital of Guangzhou University of Chinese Medicine Guangzhou China

**Keywords:** brain metastasis, exosomes, germacrone, myeloid‐derived suppressor cells, s100a14/pias3/stat3 axis

## Abstract

Brain metastasis (BrM) is the most common complication with the highest mortality in clinical tumor patients. Its underlying pathogenesis remains unclear, and effective therapeutic compounds are currently unavailable. Herein, we first revealed that S100A14 is a critical molecular mediator of tumor‐derived exosomes (EVs)‐driven BrM. DIA‐based proteomics demonstrated consistently high S100A14 expression in BrM‐EVs from both cells (53.2‐fold in A549 BM3 cells and 2.4‐fold in 4T1 BM2 cells) and clinical patient samples (5.1‐fold). Intracardiac injection mice model confirmed that S100A14‐overexpression EVs promoted BrM in breast (4T1: 88.89% vs. 44.45%; MDA‐MB‐231: 66.67% vs. 33.33%) and lung cancer (LLC: 50% vs. 33.33%; A549: 83.33% vs. 33.33%). Mechanistically, S100A14 directly targeted PIAS3 to reprogram astrocytes (AS) by activating STAT3 signaling and triggering secretion of pro‐inflammatory chemokines (CCL2/CCL5/CXCL5), which recruit immunosuppressive MDSCs. Crucially, we identified the natural compound, germacrone, as a promising therapeutic agent that effectively inhibited BrM progression in both lung and breast cancer without significant toxicity. Germacrone directly binds to S100A14 in AS to disrupt the S100A14‐PIAS3 interaction, inhibit STAT3 activation, and MDSCs recruitment. Our study elucidated a novel mechanism by which tumor‐derived S100A14 EVs promote tumor BrM. Moreover, germacrone emerged as a prospective therapeutic agent for preventing BrM by specifically targeting S100A14.

## Graphical abstract

This study identifies S100A14 in tumor‐derived exosomes as a key driver of brain metastasis. S100A14 targets PIAS3 in astrocytes, activating STAT3 signaling and promoting immunosuppressive MDSCs recruitment via chemokine secretion. Germacrone, a natural compound, binds S100A14 to disrupt this axis, effectively inhibiting brain metastasis with low toxicity.

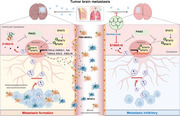

**Abbreviations**
BrM: Brain metastases
AS: Astrocytes
RAs: Reactive astrocytes
GFAP: Glial fibrillary acidic protein
STAT3: Signal Transducer and Activator of Transcription 3
PIAS: The Protein Inhibitors of Activated STAT
TCM: Traditional Chinese Medicine
GM: Germacrone
TMZ: Temozolomide
EVs: Exosomes
BM‐EVs: BrM tumor cells‐derived EVs
S100A14OE: S100A14‐overexpressing
HBSS: Hank's balanced salt solution
PDL: Poly‐D‐lysine
MACS: Magnetic‐activated cell sorting
NTA: Nanoparticle tracking check
TEM: Transmission electron microscopy
Y2H: Yeast two‐hybrid
CO‐IP: Co‐immunoprecipitation
BIFC: Bimo‐lecular fluorescence complementation
DIA: Data independent acquisition
MD: Molecular Dynamics
qRT‐PCR: Quantitative Real‐Time PCR
WB: Western Blot
CETSA: Cellular thermal shift
DARTS: Drug affinity responsive target stability
IF: Immunofluorescence
ICT: Intracardiac injection model
ICD: Intracarotid injection model
BLI: Bioluminescent intensity
MDSCs: Myeloid‐derived suppressor cells
P‐MDSCs: Polymorphonuclear myeloid‐derived suppressor cells
M‐MDSCs: Monocytic myeloid‐derived suppressor cells
LC: Lung cancer
LC‐BM: Lung cancer brain metastasis
BBB: Blood‐brain barrier
PCA: Principal component analysis
CSF: Collected cerebrospinal fluid

## 1 Introduction

Brain metastasis (BrM) is a devastating complication of advanced malignancies and a leading cause of cancer‐related mortality [1, 2, 3]. Epidemiological data reveal that up to 50% of patients with lung cancer (LC) [4, 5]and 15% to 35% of breast cancer patients develop BrM [6, 7] during cancer progression. Owing to the unique anatomy and physiological function of the brain, treatment options for patients with BrM are limited. Although recent clinical trials have demonstrated that BrM exhibits initial response rates to approved targeted and immunotherapies, these intracranial responses are often less durable and ultimately lead to mortality in most patients. Therefore, radiotherapy remains the standard treatment, and the median overall survival of patients with BrM is 4–8 months. Therefore, there is an urgent need to study the mechanisms underlying tumor BrM and develop effective treatment strategies [2, 8, 9].

Exosomes (EVs) are extracellular vesicles with a diameter of approximately 30–150 nm, and contain various bioactive substances such as proteins and nucleic acids [10, 11]. AS “micro messengers” for intercellular communication, they play a crucial role in disease progression. For instance, SP1 in 4T1‐derived EVs favors the secretion of IL‐1β by neutrophils through the activation of the TLR4‐NFκβ pathway, ultimately aggravating lung metastasis [12]. Complement C3 of renal cell carcinoma cell‐derived EVs induces the secretion of CCL2 and CXCL1 by lung macrophages and subsequently enhances tumor‐associated macrophages (TAMs) polarization and polymorphonuclear myeloid‐derived suppressor cell (P‐MDSCs) recruitment [13]. Tripartite motif‐containing 59 (TRIM59) in cancer‐derived EVs regulates macrophage NLRP3 inflammasome activation to promote lung metastasis [14]. Increasing evidence indicates that EVs secreted by tumor cells perform multiple functions in tumor BrM, ranging from preparing the pre‐metastatic microenvironment to penetrating the Blood Brain Barrier (BBB), immune evasion, and colonization of metastatic lesions, all of which exert critical metastasis effects [15, 16, 17, 18].

In particular, the pathophysiology of BrM involves a complex cascade of events, which is the ultimate outcome of the interaction between circulating tumor cells and the brain tissue microenvironment. Recent studies have elucidated the important role of astrocytes (AS), which are the most abundant glial cells in the brain, and are implicated as key components of the BBB in facilitating of BrM in various cancer types [19, 20]. Specifically, reactive AS (RAs), characterized by high levels of glial fibrillary acidic protein (GFAP), are critical regulators of BrM [21, 22, 23]. RAs interact with cancer cells in the brain and exhibit neuroinflammation characteristics, including the release of pro‐inflammatory cytokines and chemokines, increased BBB permeability, and infiltration of immune cells [24, 25, 26].

The signal transducer and activator of transcription 3 (STAT3) signaling pathway plays a crucial role in the process by which RAs shape an immunosuppressive microenvironment [27]. In clinical patients, active STAT3 in RAs is correlated with a reduced survival rate after BrM diagnosis. Studies confirmed that STAT3 activation in RAs benefits brain metastatic cells by affecting the innate and acquired immune system [24, 28]. Specifically, pSTAT3^+^ RAs not only activated microglia/macrophages through the macrophage migration inhibitory factor (MIF)‐CD74 pathway, inducing the release of tumor‐promoting factor midkine and activating innate immunity, but also secreted factors such as PD‐L1 and VEGF‐A, inhibiting the activity of CD8^+^ T cells and forming a physical barrier to hinder immune infiltration and suppress adaptive immunity [29]. Study also showed that chitosanase 3‐like protein 1 (CHI3L1) secreted by pSTAT3^+^ RAs activates the AKT signaling pathway in cancer cells and promots the invasive growth of tumor cells in brain tissue [30]. Therefore, RAs labeled with pSTAT3 have recently emerged as a promising therapeutic target for BrM.

Protein Inhibitors of Activated STAT (PIAS) represents a class of endogenous negative regulatory proteins that suppress STAT transcriptional activity via multiple mechanisms, including SUMOylation and transcriptional regulation. These proteins play crucial roles in various biological processes such as inflammatory responses and immune regulation. The PIAS family is comprised of four principal members (PIAS1, PIAS2, PIAS3, and PIAS4) that exhibit selective regulatory specificity for different STAT family members. Notably, PIAS3 specifically binds to phosphorylated STAT3, thereby blocking its DNA‐binding capacity and inhibiting transcriptional activity. AS a primary defense mechanism against STAT3 hyperactivation, PIAS3 serves critical roles in both immune regulation and cancer pathogenesis [31, 32].

S100A14 is a small molecule composed of 101 amino acids (with a molecular weight of 14 kDa) belonging to the calcium‐binding S100 protein family. It was initially identified in 2000 as a calcium‐binding protein that is expressed in various tissues [33]. Subsequent studies revealed that S100A14 is overexpressed in various cancers, including esophageal, gastric, colorectal, LC, and breast cancers, and plays a role in promoting tumor occurrence and development. However, there are currently no studies on S100A14 in relation to tumor BrM [34, 35].

In the current study, we first elucidated the role and underlying mechanisms of tumor‐derived S100A14‐enriched EVs in promoting BrM by specifically targeting PIAS3 in RAs. Clinical BrM patient samples and in vitro BrM cells consistently demonstrated significant upregulation of S100A14 protein within EVs derived from BrM. Mechanistically, we revealed that S100A14‐EVs directly bind to PIAS3 in RAs, thereby activating the STAT3 signaling pathway. The activation of STAT3 triggers a marked increase in the secretion of pro‐inflammatory chemokines, such as CCL2, CCL5, and CXCL5, which facilitates the recruitment of MDSCs, ultimately forming an immunosuppressive microenvironment, conducive to tumor BrM [36, 37, 38, 39]. Germacrone (GM), a natural sesquiterpene ketone compound, is widely present in traditional Chinese medicines (TCM), such as *Curcuma longa, Curcuma zedoaria*, and *Curcuma wenyujin*, and possesses various pharmacological effects, including anti‐inflammatory, antitumor, and neuroprotective properties [40, 41]. We further clarified that germacrone can effectively inhibit the binding of S100A14 and PIAS3, suppress the secretion of pro‐inflammatory chemokines by AS, and reprogram the brain tumor immune landscape to prevent BrM. Our findings provide insights into the molecular mechanisms through which tumor‐secreted S100A14‐EVs shape the brain immunosuppression niche to promote BrM. Germacrone has emerged as a prospective therapeutic agent for preventing BrM by specifically targeting the S100A14‐PIAS3‐STAT3 axis.

## 2 Results

### 2.1 BM‐EVs Promote BrM by Enriching GFAP^+^ RAs and Shaping Immunosuppressive Microenvironment

**FIGURE 1:**
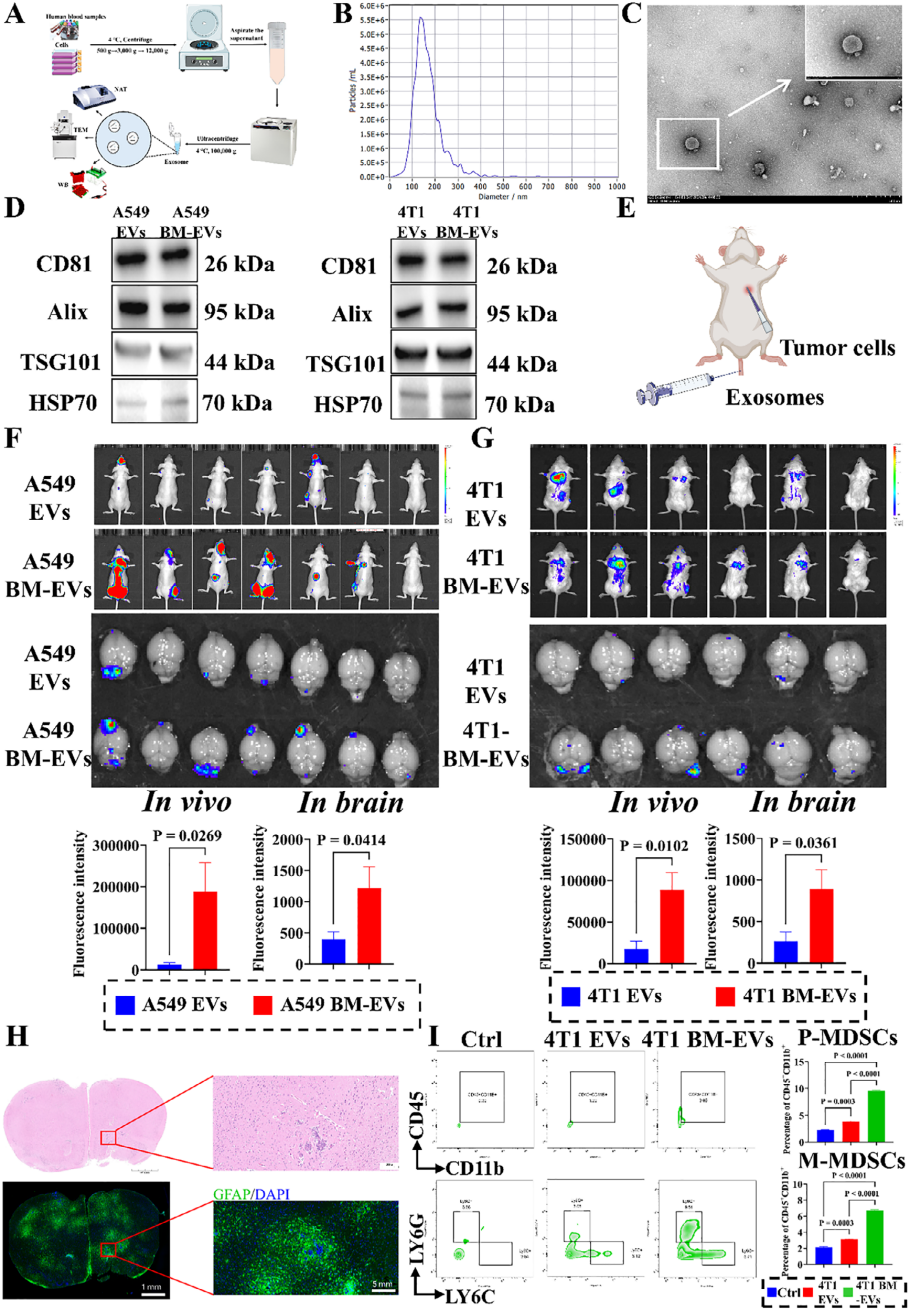
BM‐EVs promote BrM by enriching GFAP^+^ RAs and shaping immunosuppressive microenvironment. (A) EVs were isolated from cell culture medium and human blood by ultracentrifugation. (B) NTA and (C) TEM analysis of EVs. (D) Western blot probing for exosomal markers ALIX, CD81, TSG101, and HSP70. (E) Experimental flowchart of BrM model in mice, which involved intracardiac injection of tumor cells and tail vein injection of Evs. (F) In vivo and *in brain* bioluminescence imaging and quantification of brain metastatic burden in mice bearing A549 tumors following EVs. (G) In vivo and *in brain* bioluminescence imaging and quantification of brain metastatic burden in mice bearing 4T1 tumors following EVs. (H) IF staining for GFAP (green) and nucleus (blue) in brain. (I) Flow cytometry quantification of P‐MDSCs and M‐MDSCs in control brain, 4T1 EVs treatment group, and 4T1 BM‐EVs treatment group. Data represent mean ± SEM. Significant difference versus control group, ^*^ *P*<0.05, ^**^ *P*<0.01, and ^***^ *P*<0.001.

To investigate the role of EVs derived from BrM tumor cells in the process of BrM, we first used ultracentrifugation to isolate EVs from the cell culture medium and human blood (Figure 1A). Isolated EVs were characterized by NTA for size distribution, TEM for morphology, and western blotting for protein marker expression. NTA revealed that the average particle diameter of isolated EVs was 139 ± 8 nm (Figure 1B). TEM imaging confirmed the typical cup‐shaped, double‐membrane morphology of EVs (Figure 1C). Western blot analysis confirmed the presence of exosomal markers, including CD81, Alix, TSG101, and HSP70 in EVs derived from both parental (A549 and 4T1) and BrM (A549‐BM and 4T1‐BM) tumor cells (Figure 1D). These results collectively verify the successful isolation of high‐purity EVs.

To assess the functional impact of BrM tumor cell‐derived EVs (BM‐EVs) on BrM, a mouse BrM model was established by intracardiac injection of tumor cells combined with intravenous injection of EVs (Figure 1E). Notably, BM‐EVs significantly enhanced brain metastatic efficiency in both A549 LC (57.14% vs. 100%) and 4T1 breast cancer (50.00% vs. 83.33%, respectively) models. Specifically, in A549 LC model, mice treated with BM‐EVs exhibited a significantly higher brain metastatic burden compared to that in the control group, as evidenced by an increased fluorescence intensity in the body (12787.20 ± 12950.94 in EVs group vs. 188367.96 ± 183829.29 in BM‐EVs group, p = 0.0269) and brain (391.81 ± 339.05 in EVs group vs. 1217.29 ± 894.20 in BM‐EVs group, p = 0.0414) (Figure 1F). Similarly, in the 4T1 breast cancer model, BM‐EVs also markedly increased fluorescence intensity both in vivo and in the brain (in vivo: 17266.60 ± 14586.54 vs. 88932.20 ± 49807.12, p = 0.0102; in the brain: 264.46 ± 175.12 vs. 891.10 ± 571.47, p = 0.0361) (Figure 1G). These results demonstrated that BM‐EVs significantly facilitated tumor BrM.

IF staining of the brain tissue revealed significant enrichment of GFAP^+^ RAs surrounding the metastatic tumor foci in mice traversed with BM‐EVs (Figure 1H). Furthermore, flow cytometry analysis of brain‐infiltrating immune cells demonstrated a substantial increase in the number of MDSCs in BrM‐treated mice. Specifically, the proportions of P‐MDSCs (CD45^+^CD11b^+^Ly6G^+^) and M‐MDSCs (CD45^+^CD11b^+^Ly6C^+^) were elevated compared to those in normal control (P‐MDSCs: 2.27% ± 0.09% vs. 3.81% ± 0.23%; M‐MDSCs: 2.18% ± 0.09% vs. 3.12% ± 0.11%) (Figure 1I). More importantly, the administration of BM‐EVs further amplified this immunosuppressive landscape, resulting in a greater infiltration of both P‐MDSCs (3.81% ± 0.23% vs. 9.57% ± 0.27%, p < 0.001) and M‐MDSCs (3.12% ± 0.11% vs. 6.71% ± 0.18%, p < 0.001) compared to that in the group receiving EVs from tumor cells. In summary, our findings demonstrate that BM‐EVs enhance BrM by fostering a permissive microenvironment characterized by the enrichment of GFAP^+^ RAs and the recruitment of immunosuppressive MDSCs populations.

### 2.2 S100A14 as a Key Molecular Mediator of BM EVs‐Driven Microenvironment Remodeling in BrM

**FIGURE 2:**
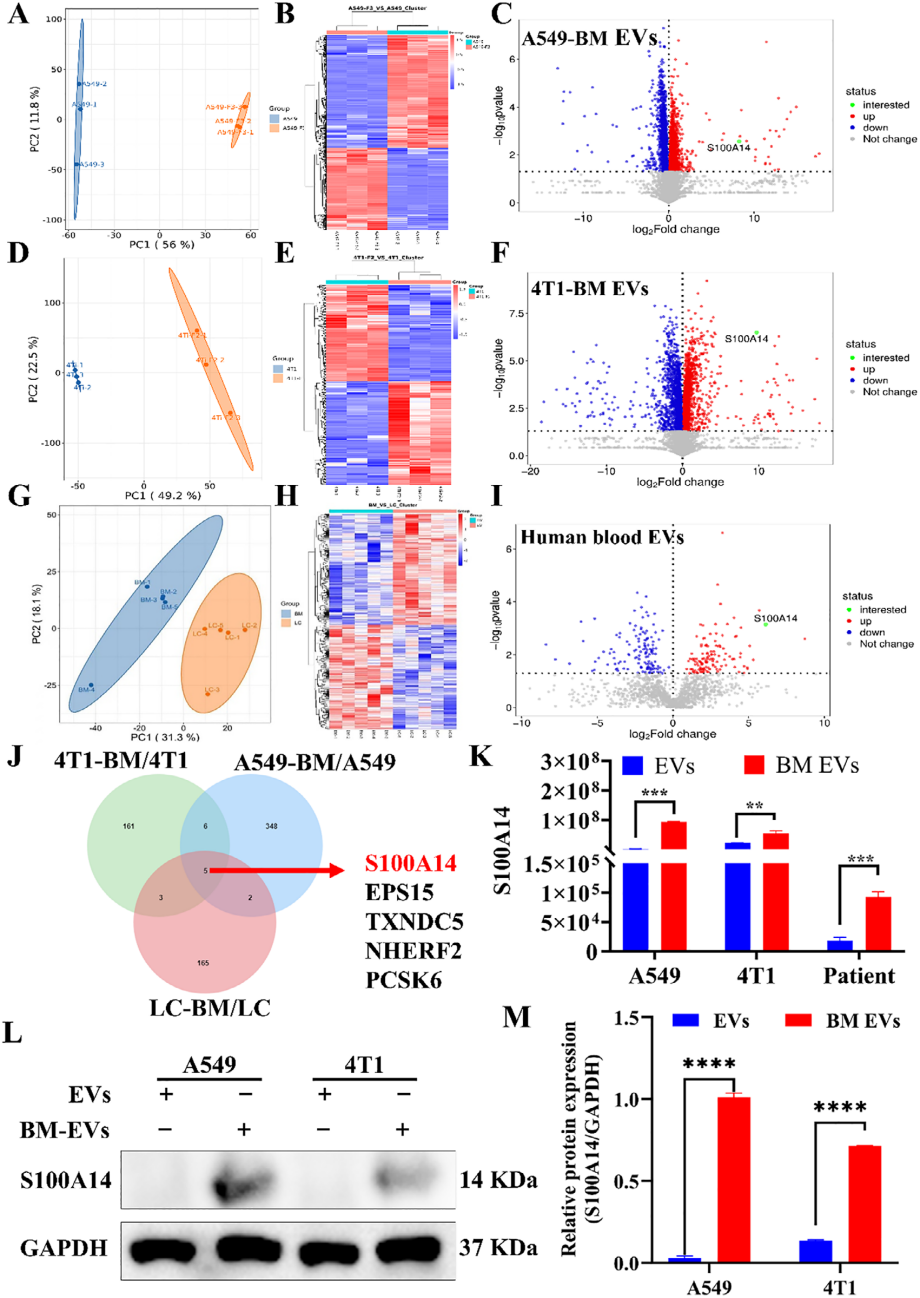
S100A14 as a key molecular mediator of BM EVs‐driven microenvironment remodeling in BrM. (A) PCA of A549 EVs and A549 BM‐EVs proteomics. Each dot represents a sample, and samples are coloured by group. (B) Heatmap of differentially expressed proteins in A549 EVs and A549 BM‐EVs. (C) Volcano plot of differentially expressed proteins in A549 EVs and A549 BM‐EVs. (D) PCA of 4T1 EVs and 4T1 BM‐EVs proteomics. Each dot represents a sample, and samples are coloured by group. (E) Heatmap of differentially expressed proteins in 4T1 EVs and 4T1 BM‐EVs. (F) Volcano plot of differentially expressed proteins in 4T1 EVs and 4T1 BM‐EVs. (G) PCA of LC patient EVs and LC‐BM patient EVs proteomics. Each dot represents a sample, and samples are coloured by group. (H) Heatmap of differentially expressed proteins in LC patient EVs and LC‐BM patient EVs. (I) Volcano plot of differentially expressed proteins in LC patient EVs and LC‐BM patient EVs. (J) Venn diagram representing the overlap among the differentially regulated proteins quantified in A549‐BM EVs, 4T1‐BM EVs, and LC‐BM EVs. (K) S100A14 levels in EVs extracted in lung cancer cells, breast cancer cells, and clinical lung cancer patients. Protein (L) and mRNA (M) expression levels of S100A14 in EVs and BM‐EVs. Data represent mean ± SEM. Significant difference versus control group, ^*^ *P*<0.05, ^**^ *P*<0.01, and ^***^ *P*<0.001.

To explore the key proteins within BM‐EVs that drive BrM, we performed DIA‐based quantitative proteomic profiling to comprehensively analyze EVs and BM‐EVs. Principal component analysis (PCA) revealed a distinct separation along the PC1 axis between parental EVs and BM‐EVs in both A549 (Figure 2A) and 4T1 (Figure 2D) cancer cells, indicating significant global differences in their protein expression profiles. The tight clustering of biological replicates within each group suggests high reproducibility and reliable data quality. Heatmaps (Figure 2B,E) and volcano plots (Figure 2C,F) also showed significant differences in protein expression between the parental EVs and BM‐EVs. Specifically, compared with that in parental EVs, 361 proteins were significantly upregulated and 534 were downregulated in A549 BM‐EVs (Figure 2C), whereas 175 proteins were upregulated and 162 were downregulated in 4T1 BM‐EVs (Figure 2F). To assess the clinical relevance of the proteins identified in BM‐EVs, we performed quantitative proteomics on serum‐derived EVs from patients with LC and brain metastasis (LC‐BM). PCA (Figure 2G) and heatmap (Figure 2H) analyses showed significant differences in protein expression between the serum EVs of the primary LC and LC‐BM patient groups. Notably, 175 proteins were significantly upregulated and 189 were downregulated in EVs from patients with LC‐BM compared to those from patients with LC (Figure 2I). Intersectional analysis of BM‐EVs isolated from cells and clinical patient blood identified five proteins that were consistently upregulated in A549‐BM EVs, 4T1‐BM EVs, and LC‐BM EVs: S100A14, EPS15, TXNDC5, NHERF2, and PCSK6 (Figure 2J). Among these candidates, S100A14 is particularly noteworthy. Mass spectrometry data confirmed that S100A14 was significantly elevated in A549‐BM EVs (53.19 ± 1.25‐fold higher), 4T1‐BM EVs (2.38 ± 0.70‐fold higher), and LC‐BM EVs (5.10 ± 1.03‐fold higher) compared to those in the control group EVs (Figure 2K). Subsequently, western blotting was used to detect the protein expression of S100A14 in A549‐BM and 4T1‐BM EVs. The results showed that S100A14 protein expression in BM‐EVs was significantly higher than that in parental EVs (Figure 2L). Quantitatively, S100A14 protein expression levels were increased by 35.15 ± 1.46‐fold in A549‐BM EVs and 5.30 ± 0.02‐fold in 4T1‐BM EVs compared to that in their corresponding parental cell‐derived EVs (Figure 2M). In conclusion, our integrated proteomic and validation analyses identified that S100A14 is highly expressed in BM‐EVs, highlighting its potential as a critical effector in driving the brain metastatic cascade.

### 2.3 S100A14^OE^ EVs Significantly Promote BrM by Enriching GFAP^+^ RAs and Shaping the Immunosuppressive Microenvironment

To elucidate the role and mechanism of S100A14^OE^ EVs in promoting tumor BrM, we first established stable S100A14‐overexpressing (S100A14^OE^) cell lines derived from LC (A549 and LLC) and breast cancer (MDA‐MB‐231 and 4T1) cells via lentiviral transduction. Western blotting and qRT‐PCR analyses confirmed that both S100A14 protein and mRNA levels were significantly upregulated (Figure S1A,B). Subsequently, EVs from parental and S100A14^OE^ cells were isolated and characterized by NTA, TEM, and western blotting (Figure S1C,D). Consistently, S100A14 protein levels in S100A14^OE^ EVs were also significantly elevated by approximately 4.04 ± 0.19‐fold in A549‐S100A14^OE^, 6.40 ± 0.28‐fold in LLC‐S100A14^OE^, 3.99 ± 0.29‐fold in MDA‐MB‐231‐S100A14^OE^, and 2.09 ± 0.05‐fold in 4T1‐S100A14^OE^ EVs compared to those in EVs from their parental cells (Figure S1E).

**FIGURE 3:**
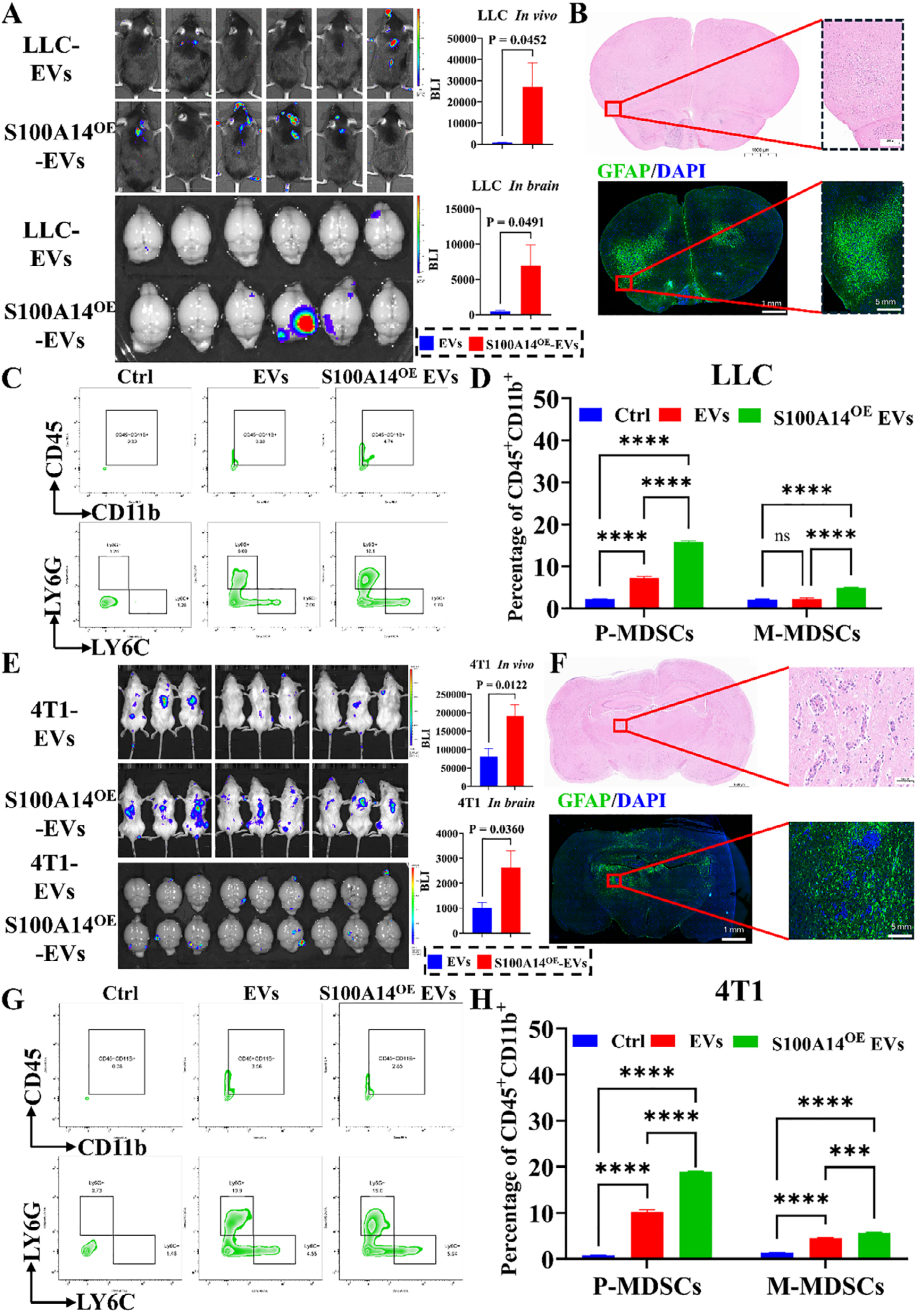
S00A14^OE^ EVs significantly promote BrM by enriching GFAP^+^ RAs and shaping the immunosuppressive microenvironment. (A) In vivo and *in brain* bioluminescence imaging and quantification of brain metastatic burden in mice bearing LLC tumors following S00A14^OE^ EVs. (B) The HE staining and GFAP IF staining of brain tissue. (C) Flow cytometry analysis of MDSCs in brain of mice from control, LLC EVs, or LLC S00A14^OE^ EVs treatment groups. (D) The quantification of P‐MDSCs and M‐MDSCs in control, LLC EVs, or LLC S00A14^OE^ EVs treatment groups. (E) In vivo and *in brain* bioluminescence imaging and quantification of brain metastatic burden in mice bearing 4T1 tumors following S00A14^OE^ EVs. (F) The HE staining and GFAP IF staining of brain tissue. (G) Flow cytometry analysis of MDSCs in brain of mice from control, 4T1 EVs, or 4T1 S00A14^OE^ EVs treatment groups. (H) The quantification of P‐MDSCs and M‐MDSCs in control, LLC EVs, or LLC S00A14^OE^ EVs treatment groups. Data represent mean ± SEM. Significant difference versus control group, ^*^ *P*<0.05, ^**^ *P*<0.01, and ^***^ *P*<0.001.

Finally, an ICT mouse model was used to validate the pro‐metastatic function of S100A14^OE^ EVs in vivo. In LC, S100A14^OE^‑EVs significantly enhanced the incidence of BrM in both A549 (83.33% vs. 33.33%) and LLC (50.00% vs. 33.33%) cell‐derived ICT models. Quantitative analysis of BLF (bioluminescence intensity) demonstrated that S100A14^OE^ EVs not only markedly increased BLF in vivo (695.83 ± 336.03 in EVs group vs 28185.00 ± 20018.33 in S100A14^OE^ EVs group, p = 0.0452), but also significantly enhanced BLF in the brain (487.33 ± 309.11 in EVs group vs 6971.5 ± 5083.37 in S100A14^OE^ EVs group, p = 0.0491) in LLC‐bearing mice (Figure 3A). Similar results were observed in A549‐bearing mice (Figure S2A,C). This pro‐metastatic effect was recapitulated in breast cancer models, where S100A14^OE^‑EVs significantly increased BLF in vivo (81145.50 ± 66309.62 in EVs group vs 198884.00 ± 94609.62 in S100A14^OE^ EVs group, p = 0.0122) and in the brain (1021.11 ± 508.88 in EVs group vs 2633.84 ± 1323.21 in S100A14^OE^ EVs group, p = 0.0360) in 4T1‐bearing mice (Figure 3E). S100A14^OE^‑EVs also significantly increased BLF in MDA‐MB‐231‐bearing mice (Figure S2B,D). These results collectively confirm that S100A14^OE^‑EVs potently promote BrM of both lung and breast cancer cells.

To investigate the underlying mechanisms, we performed HE and IF staining on brain tissues, which revealed a striking accumulation of GFAP^+^ RAs surrounding metastatic lesions in the S100A14^OE^‑EVs treatment group in both lung (Figure 3B; Figure S2E) and breast (Figure 3F; Figure S2F) cancer models. Flow cytometric analysis of brain‐infiltrating immune cells demonstrated that S100A14^OE^‑EVs treatment consistently increased the infiltration of MDSCs in the brain tissue of LLC‐bearing mice (P‐MDSC, 7.27% ± 0.61% vs 15.77% ± 0.61%, p < 0.001; M‐MDSC, 2.29% ± 0.33% vs 4.92% ± 0.14%, p < 0.001) and 4T1‐bearing mice (P‐MDSC, 10.30% ± 0.63% vs 18.95% ± 0.17%, p < 0.001; M‐MDSC, 4.56% ± 0.11% vs 5.70% ± 0.16%, p < 0.001) (Figure 3C,D,G,H). Taken together, our results demonstrate that S100A14^OE^‑EVs enhance BrM by promoting aggregation of GFAP^+^ RAs to tumor lesions and shaping the immunosuppressive microenvironment, characterized by increased MDSCs infiltration in the brain.

### 2.4 S100A14^OE^ EVs Promote BrM by Reprogramming AS Via STAT3 Signaling to Induce Pro‐Inflammatory Chemokines Secretion and Recruit MDSCs

**FIGURE 4:**
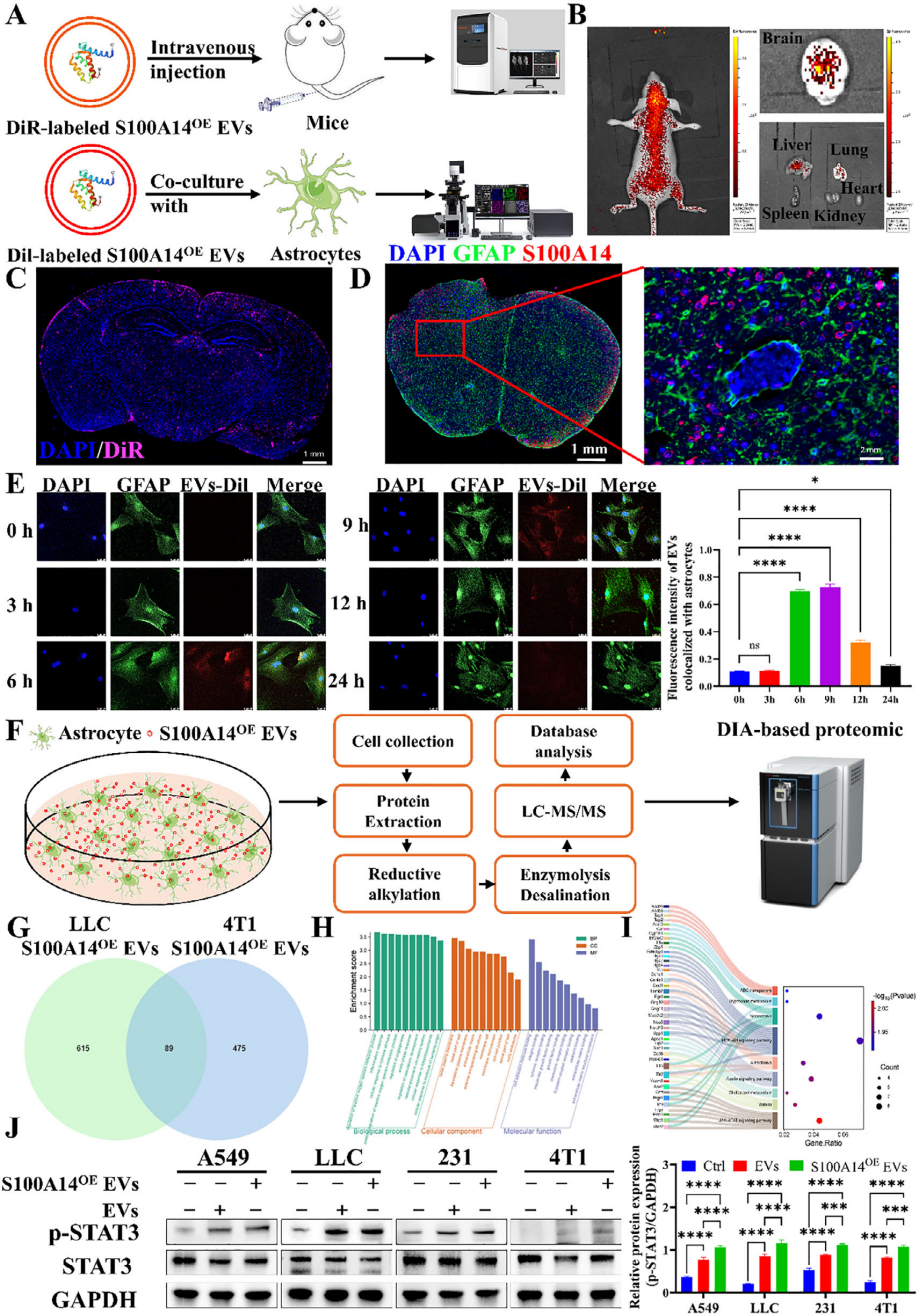
S100A14^OE^ EVs promote BrM by reprogramming AS via STAT3 signaling pathway. (A) Schematic diagram of S100A14^OE^ EVs stained with Dil and DiR. (B) The in vivo fluorescence imaging and organ distribution of mice after tail vein injection of DiR‐labeled S100A14^OE^ EVs. (C) IF detection of brain tissue treated with DiR‐labeled S100A14^OE^ EVs. (D) GFAP IF staining was performed on brain tissue from the S100A14^OE^ EVs treatment group. (E) Schematic diagram and statistical analysis of the uptake of Dil‐labeled S100A14^OE^ EVs by astrocytes. (F) Schematic diagram of DIA proteomic analysis about S100A14^OE^ EVs co‐culture with astrocytes. (G) A Venn diagram showed upregulated proteins between LLC S100A14^OE^ EVs co‐cultured astrocytes and 4T1 S100A14^OE^ EVs co‐cultured astrocytes. (H) Top 20 (ranked by p‐value) GO enrichment analyses of three categories (BP: biology process, CC: cell component, MF: molecular function). (I) Top 20 KEGG pathways enrichment analyses. (J) Protein expression levels and quantification of p‐STAT3 and STAT3 in AS treated with S100A14^OE^ EVs. Data represent mean ± SEM. Significant difference versus control group, ^*^ *P*<0.05, ^**^ *P*<0.01, and ^***^ *P*<0.001.

To further explore the interaction between S100A14^OE^ EVs and AS, we first prepared DiR‐labeled EVs for in vivo experiments and Dil‐labeled EVs for cell experiments (Figure 4A). After intravenous injection of DiR‐labeled EVs, the distribution of EVs was observed throughout the whole body, especially in the brain, which revealed that DiR‐labeled EVs could achieve in vivo visualization after administration (Figure 4B). IF staining confirmed that DiR‐labeled EVs could cross the BBB and accumulate in the brain tissue (Figure 4C). Importantly, IF staining of brain tissue in the ICT model demonstrated that S100A14^OE^ EVs co‐localized with GFAP^+^ RAs and were highly enriched around tumor cells (Figure 4D). These results imply that there is complex crosstalk among S100A14^OE^ EVs, GFAP^+^ RAs, and tumor cells. Subsequently, an uptake experiment of AS was conducted to verify the above. AS shown in Figure 4E, after co‐culture for 6 h, strong co‐localization of S100A14‐astrocyte fluorescence intensity (10.96% ± 0.24% compared to 69.91% ± 0.95%, p < 0.001) could be observed, and the fluorescence intensity decreased over time. Therefore, 6 h of co‐culture was chosen for subsequent DIA proteomic analysis to investigate the targets and identify signaling pathways (Figure 4F). Our results showed that after treatment with S100A14^OE^ EVs, 704 proteins were significantly upregulated in LLC S100A14^OE^ EVs‐treated AS group, and 564 proteins were significantly upregulated in 4T1 S100A14^OE^ EVs‐treated AS group (Figure 4G). GO and KEGG pathway enrichment analyses of these differentially expressed proteins further revealed biological processes and signaling pathways (Figure 4H,I). These biological processes and signaling pathways include the JAK‐STAT, PI3K‐AKT, necroptosis, and apelin signaling pathways. Western blot results demonstrated that S100A14^OE^ EVs treatment significantly activated STAT3 in the AS group, which significantly increased the expression of p‐STAT3 (Figure 4J). These results confirmed that S100A14^OE^ EVs activated the STAT3 signaling pathway after being taken up by AS.

**FIGURE 5:**
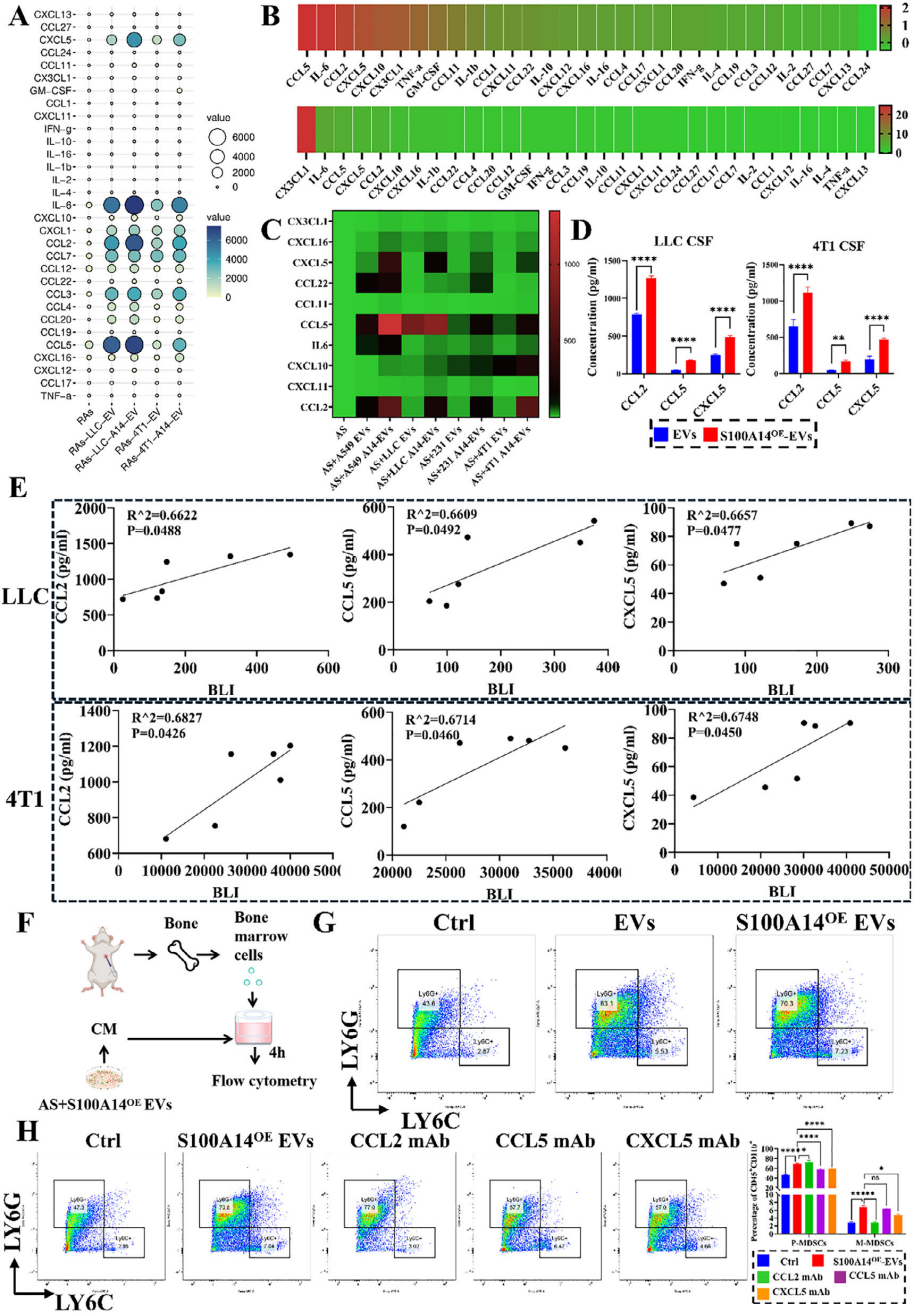
S100A14^OE^ EVs reprogram AS to induce pro‐inflammatory chemokines secretion and MDSCs recruitment. (A) Luminex multiplex assay to quantify the secretion of 31 cytokines from the supernatant of AS treated with EVs and S100A14^OE^ EVs. (B) Heat map of the fold changes of 31 cytokines in the supernatant of AS cells treated with S100A14^OE^ EVs compared to EVs. (C) Heat map of the mRNA expressions of the top 10 cytokines in the supernatant of AS cells treated with S100A14^OE^ EVs compared to EVs. (D) ELISA results for CCL2, CCL5, and CXCL5 in CSF of LLC tumor‐bearing and 4T1 tumor‐bearing mice. (E) Correlation analysis of chemokines (CCL2, CCL5, CXCL5) in CSF and brain metastasis. (F) Schematic diagram of ex vivo MDSC recruitment assay. (G) Flow cytometry analysis of MDSCs in control, EVs treatment, and S00A14^OE^ EVs treatment. (H) Flow cytometry analysis of P‐MDSCs and M‐MDSCs in control, S00A14^OE^ EVs treatment, S00A14^OE^ EVs+CCL2mAb treatment, S00A14^OE^ EVs+CCL5mAb treatment, and S00A14^OE^ EVs+CXCL5mAb treatment. Data represent mean ± SEM. Significant difference versus control group, ^*^ *P*<0.05, ^**^ *P*<0.01, and ^***^ *P*<0.001.

An increasing number of studies have confirmed that AS regulates cytokine release through the STAT signaling pathway. Therefore, we performed a Luminex multiplex assay to quantify the secretion of 31 cytokines from the supernatant of AS‐treated S100A14^OE^ EVs. Compared with that by control EVs, S100A14^OE^ EVs significantly upregulated the secretion of multiple cytokines (Figure 5A). Specifically, LLC‐S100A14^OE^ EVs markedly increased by 2.1‐fold in CCL5, 2.0‐fold in IL6, 1.8‐fold in CCL2, 1.6‐fold in CXCL5 and 1.4‐fold in CX3CL10. While, 4T1‐S100A14^OE^ EVs significantly elevated in 24.7‐fold in CX3CL1, 5.8‐fold in IL6, 4.6‐fold in CCL5, 3.9‐fold in CXCL5 and 3.8‐fold in CCL2 (Figure 5B). Consistent with the protein level of cytokines, qRT‐PCR analysis confirmed that S100A14^OE^ EVs from both lung and breast cancer cells significantly upregulated the mRNA expression of CCL2, CCL5, CXCL5, CXCL10, and IL6 in AS (Figure 5C). To assess the relevance of these chemokines in BrM, we collected cerebrospinal fluid (CSF) from mice with BrM and conducted a correlation analysis between cytokine levels and brain metastatic burden. ELISA results showed that in the LLC‐S100A14^OE^ EVs group, the key chemokines CCL2 (781.71 ± 51.09 compared to 1266.57 ± 71.15, p < 0.001), CCL5 (48.25 ± 6.19 compared to 186.01 ± 10.75, p < 0.001), and CXCL5 (243.75 ± 41.56 compared to 489.75 ± 34.64, p < 0.001) were significantly elevated in the CSF (Figure 5D). Correlation analysis revealed a strong positive association between the chemokines and BrM (correlation coefficient: CCL2, R^2^ = 0.6622; CCL5, R^2^ = 0.6609; CXCL5, R^2^ = 0.6657 (Figure 5E). Consistent results were observed in the 4T1‐S100A14^OE^ EVs group with parallel increases in chemokine levels (Figure 5D) and a significant correlation with breast cancer brain metastasis (Figure 5E). Given the established role of chemokines in immune cell migration, we performed a Transwell migration assay to confirm the recruitment of MDSCs. Conditioned medium from AS treated with S100A14^OE^ EVs significantly promoted the migration of both P‐MDSCs (44.3% ±1.84% compared to 68.65% ±2.19%, p < 0.001) and M‐MDSCs (2.96% ± 0.09% compared to 6.75% ± 0.45%, p < 0.001) (Figure 5G). To confirm the recruitment of chemokines, we used neutralizing antibodies against CCL2, CCL5, and CXCL5. We found that inhibition of CCL2 significantly inhibited the recruitment effect of S100A14^OE^ EVs on M‐MDSCs (6.75% ± 0.45% VS 2.89% ± 0.19%, p < 0.05). While P‐MDSC migration was potently inhibited by CCL5 inhibitor (68.65% ±2.19% VS 58.24% ± 0.98%, p < 0.001). Notably, the CXCL5 inhibitor not only could inhibit the recruitment effect of S100A14^OE^ EVs on M‐MDSCs (6.75% ± 0.45% VS 4.83% ± 0.18%, p < 0.05), but also could inhibit the recruitment effect of S100A14^OE^ EVs on P‐MDSCs (68.65% ±2.19% VS 59.2% ± 2.17%, p < 0.001) (Figure 5H). In conclusion, S100A14^OE^ EVs were reprogrammed via STAT3 activation to secrete pro‐inflammatory chemokines that recruit MDSCs.

### 2.5 S100A14 Directly Binds to PIAS3 and Actives STAT3 Signaling Pathway in AS to Induce Pro‐Inflammatory Chemokines Secretion

**FIGURE 6:**
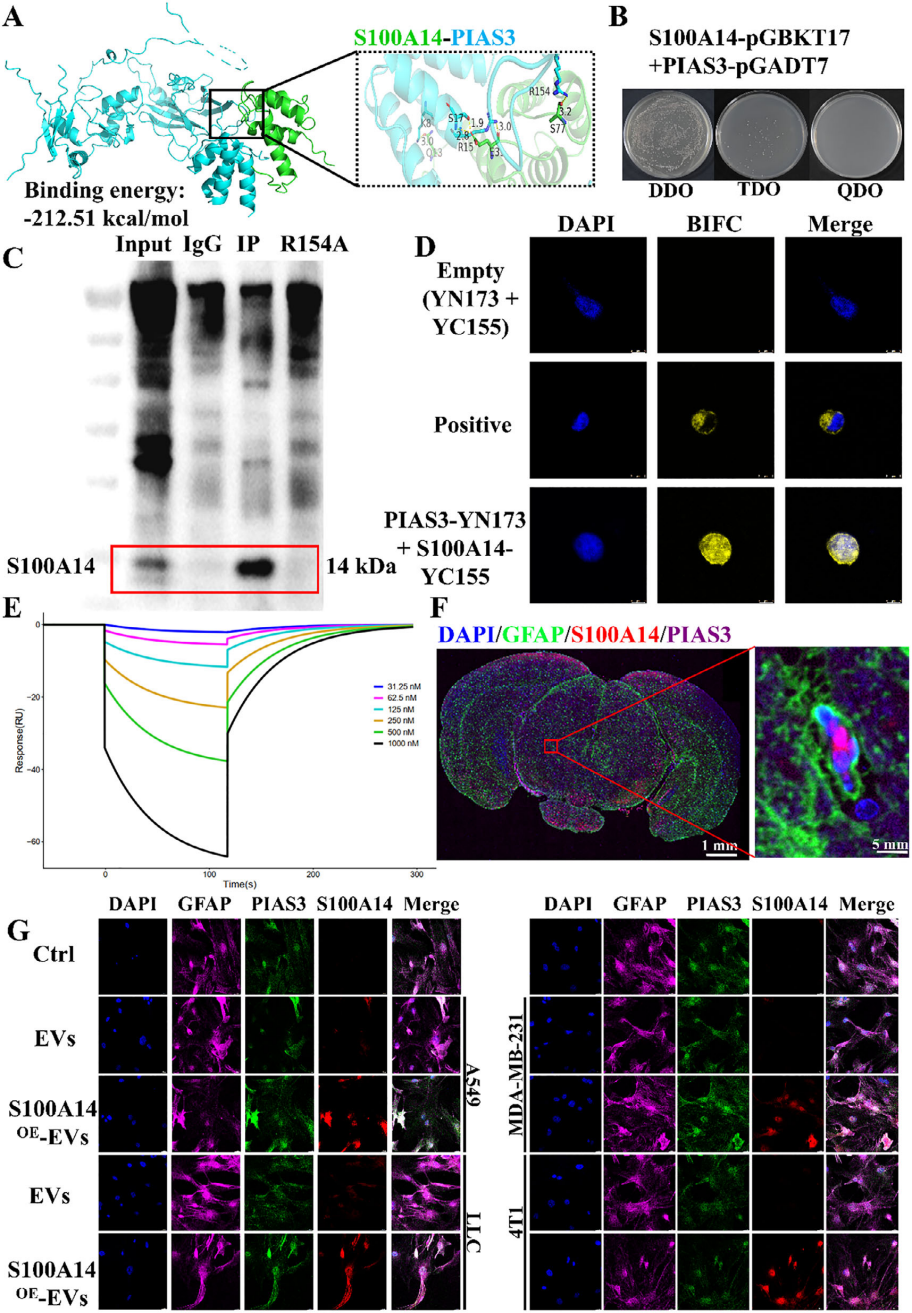
S100A14 directly binds to PIAS3 in AS. (A) The 3D diagrams of S100A14 and PIAS3 molecular docking results. (B) Results of Y2H assays between S100A14 and PIAS3. (C) CO‐IP results of S100A14, wild‐type PIAS3, and R154A mutation PIAS3. (D) BIFC assay of S100A14 and PIAS3. (E) SPR analysis of binding affinity between S100A14 and PIAS3 protein. (F) IF co‐localization results of PIAS3 and S100A14 in brain tissue. (G) IF co‐localization results of PIAS3 and S100A14 in AS treated with EVs or S00A14^OE^ EVs isolated from A549, LLC, MDA‐MB‐231, and 4T1 cells. Data represent mean ± SEM. Significant difference versus control group, ^*^ *P*<0.05, ^**^ *P*<0.01, and ^***^ *P*<0.001.

The activation process of STAT is regulated by naturally existing intracellular PIAS in normal cells, which not only binds to activated STATs to inhibit their transcriptional activity, but also promotes their SUMOylation. Our previous results showed that S100A14^OE^ EVs induced sustained activation of STAT3 upon uptake by AS. To further explore the direct target of S100A14 in AS, we first used molecular docking to predict the interaction between S100A14 and PIAS. Initial molecular docking revealed that S100A14 binds to PIAS (Figure S3A–C), with the highest affinity predicted for PIAS3 (binding energy: −212.51 kcal/mol), stabilized by hydrogen bonds (S77 and R154) and hydrophobic interactions (Figure 6A). The Y2H assay indicated a robust interaction between S100A14 and PIAS3, as evidenced by growth on selective medium and a positive β‐galactosidase activity assay (Figure 6B). The CO‐IP results showed that S100A14 specifically bound to PIAS3, which was markedly diminished when residue 154 of PIAS3 was mutated (Figure 6C). Importantly, mutation of residue 154 in PIAS3 significantly weakened or abolished this direct interaction (Figure 6C), suggesting that this site is critical for the S100A14–PIAS3 association. BIFC assays visualized strong fluorescence in living cells, confirming direct in situ evidence of interaction between S100A14 and PIAS3 (Figure 6D). SPR analysis was conducted for further quantitative analysis. The results showed that the association rate constant (Ka) of S100A14 to PIAS3 was 7.822 × 102 M^−^^1^s^−^^1^, and the dissociation rate constant (Kd) was 2.145 × 10^−^^2^ s^−^^1^ (Figure 6E). The calculated equilibrium dissociation constant was 27.42 µm, indicating that there was a high‐affinity and stable interaction between S100A14 and PIAS3, which was consistent with our CO‐IP and BiFC results. IF staining of the brain tissue revealed extensive co‐localization of S100A14 and PIAS3 in GFAP^+^ RAs in the peritumoral region (Figure 6F). Subcellular co‐localization IF analysis in AS also demonstrated that S100A14^OE^ EVs were taken up by AS and localized within the cytoplasm, where they overlapped extensively with PIAS3, which is predominantly distributed in the extra‐nuclear compartment (Figure 6G). Quantitative analysis confirmed that a high degree of co‐localization in both A549 S100A14^OE^ EVs‐treated (73.3% ± 4.2%) and 4T1 S100A14^OE^ EVs‐treated (81.0% ± 2.6%) groups, indicating that S100A14 and PIAS3 interacted and co‐localized outside the nucleus of AS. These results confirm that PIAS3 is a direct target of S100A14 in AS.

**FIGURE 7:**
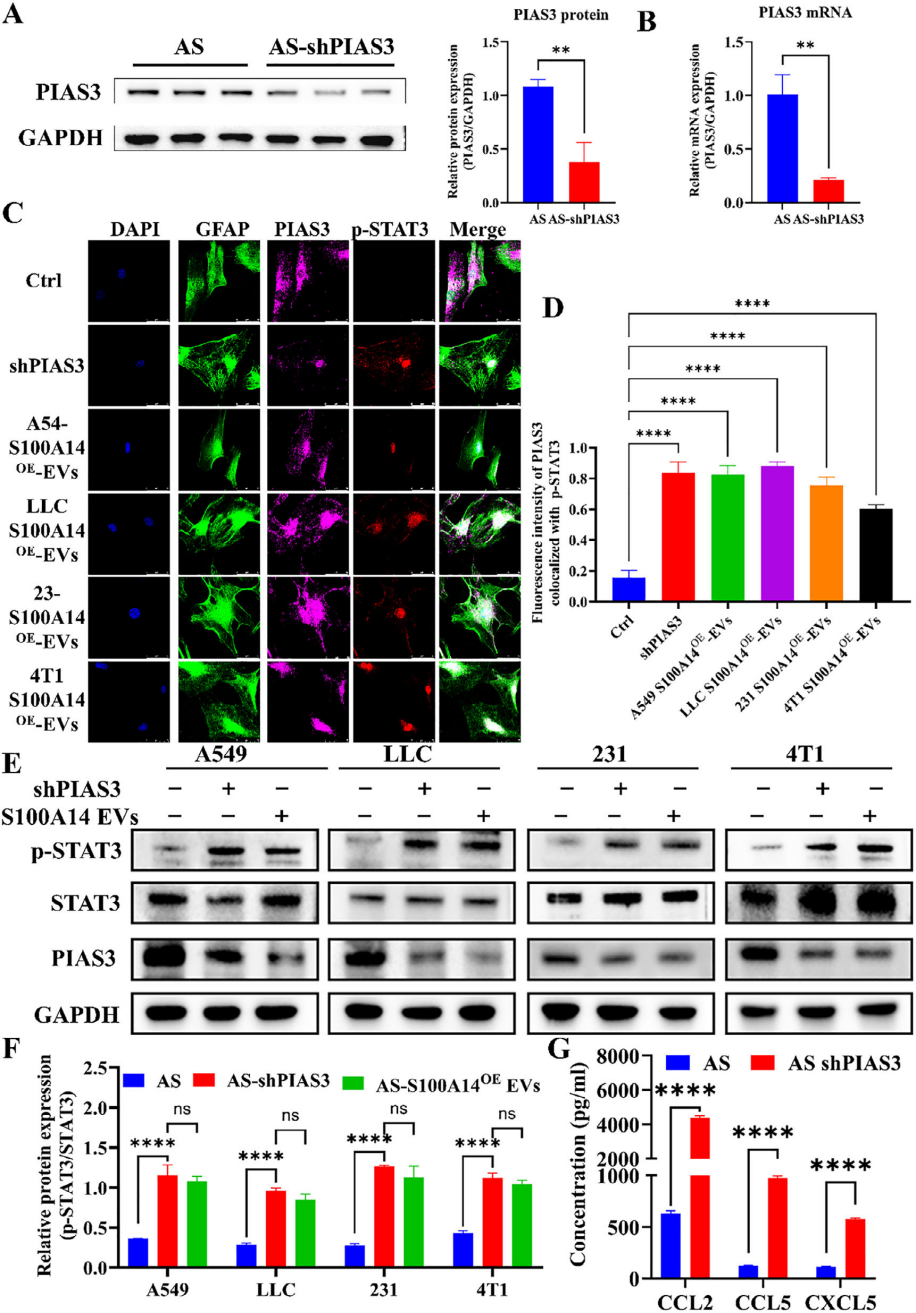
S100A14 directly binds to PIAS3 to activate the STAT3 signaling pathway in AS to induce pro‐inflammatory chemokines secretion. (A) PIAS3 protein expression in primary AS using PIAS3 knockdown plasmid transient transfection. (B) PIAS3 mRNA expression in primary AS using PIAS3 knockdown plasmid transient transfection. (C) IF co‐localization results of PIAS3 and S100A14 in AS knocked down group and AS treated with S00A14^OE^ EVs isolated from A549, LLC, MDA‐MB‐231, and 4T1 cells groups. (D) Quantitative analysis of co‐localization results of PIAS3 and S100A14 in AS knocked down group and AS treated with S00A14^OE^ EVs isolated from A549, LLC, MDA‐MB‐231, and 4T1 cells groups. Protein expression levels (E) and quantification (F) of p‐STAT3 and STAT3 in AS knocked down group and AS treated with S100A14^OE^ EVs groups. (G) ELISA results for CCL2, CCL5, and CXCL5 in the supernatant of AS‐knockdown cells. Data represent mean ± SEM. Significant difference versus control group, ^*^ *P*<0.05, ^**^ *P*<0.01, and ^***^ *P*<0.001.

To confirm that PIAS3 is a functional target of S100A14, we knocked down PIAS3 in primary AS cells. Western blotting and qRT‐PCR results showed that the protein (Figure 7A,B) and mRNA expression levels of PIAS3 in primary AS were significantly reduced following PIAS3 knockdown. IF staining of AS showed that PISA3 protein was highly expressed in GDAP^+^ As and p‐STAT3 protein was absent. In contrast, PIAS3 knockdown resulted in a marked increase in both cytoplasmic and nuclear p‐STAT3 fluorescence intensities within GFAP^+^ RAs, indicating robust activation of the STAT3 pathway. Notably, the treatment of GFAP^+^ RAs with S100A14^OE^ EVs produced effects similar to those observed following PIAS3 knockdown. Quantitative analysis of colocalization between PIAS3 and p‐STAT3 showed a significant increase in S100A14^OE^ EV‐treated (A549 S100A14^OE^ EVs: 82.8% ± 5.5%; LLC S100A14^OE^ EVs: 88.0% ± 2.8%; MAD‐MB‐231 S100A14^OE^ EVs: 75.3% ± 5.6%; 4T1 S100A14^OE^ EVs: 60.2% ± 2.8%) groups compared to that in controls (15.6% ± 4.8%) (Figure 7C,D). Consistent with the above findings, western blot analysis confirmed that S100A14^OE^ EVs treatment significantly decreased PIAS3 protein expression while concurrently increasing p‐STAT3 expression in AS (Figure 7E,F). ELISA results showed that PIAS3 knockdown could significantly increase the secretion of pro‐inflammatory chemokines CCL2 (630.4 ± 27.2 compared to 4385.1 ± 126.7, p < 0.001), CCL5 (126.1 ± 5.4 compared to 975.3 ± 22.8, p < 0.001), and CXCL5 (114.8 ± 5.6 compared to 578.9 ± 11.3, p < 0.001) in GDAP^+^ AS (Figure 7G). Finally, AS were isolated from the BrM mice using magnetic bead sorting (Figure S2G) and detected by western blotting. In summary, these findings confirm for the first time that extracellular S100A14^OE^ EVs are uptaken by AS and modulate the PIAS3‐STAT3 axis to induce the secretion of pro‐inflammatory chemokines.

### 2.6 GM Simultaneously Inhibited BrM of Breast and Lung Cancer Cells

**FIGURE 8:**
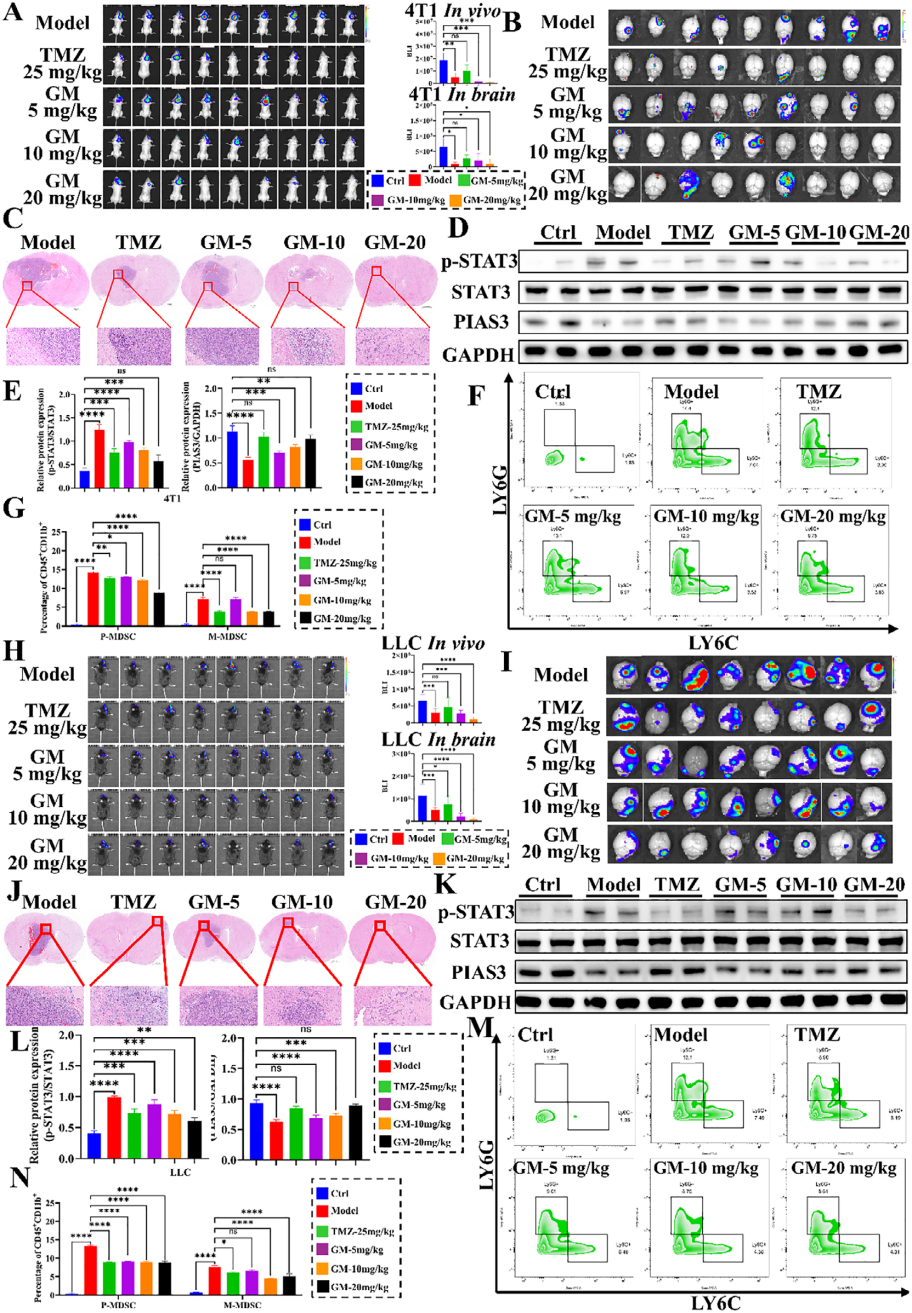
GM simultaneously inhibited BrM of breast and lung cancer cells. In vivo (A) and *in brain* (B) bioluminescence imaging and quantification of brain metastatic burden in mice bearing 4T1 tumors following S00A14^OE^ EVs. (C) The HE staining of brain tissue in model, TMZ, and different concentrations of GM treatment groups in mice bearing 4T1 tumors following S00A14^OE^ EVs. Protein expression levels (D) and quantification (E) of p‐STAT3 and STAT3 in AS isolated from mice bearing 4T1 tumors following S00A14^OE^ EVs. Flow cytometry analysis (F) and quantification (G) of P‐MDSCs and M‐MDSCs in control, TMZ, and different concentrations of GM treatment groups. In vivo (H) and *in brain* (I) bioluminescence imaging and quantification of brain metastatic burden in mice bearing LLC tumors following S00A14^OE^ EVs. (J) The HE staining of brain tissue in model, TMZ, and different concentrations of GM treatment groups in mice bearing LLC tumors following S00A14^OE^ EVs. Protein expression levels (K) and quantification (L) of p‐STAT3 and STAT3 in AS isolated from mice bearing LLC tumors following S00A14^OE^ EVs. Flow cytometry analysis (M) and quantification (N) of P‐MDSCs and M‐MDSCs in control, TMZ, and different concentrations of GM treatment groups. Data represent mean ± SEM. Significant difference versus control group, ^*^ *P*<0.05, ^**^ *P*<0.01, and ^***^ *P*<0.001.

To evaluate the in vivo efficacy of GM against tumor BrM, we administered a tumor cell intracardiac injection combined with an intravenous injection of EVs in a mouse model. In the 4T1 BrM model, BLI revealed intense metastatic signals in the brains of the control group at 13 days post‐inoculation, concomitant with significant body weight loss (>20%; Figure S4A). BLI signals in both in vivo (10 mg/kg: 1492890.9 ± 1031256.2; 20 mg/kg: 711853.3 ± 593256.9 vs. model: 18801816.0 ± 5750980.1; P<0.05) and *in brain* (10 mg/kg: 19717.3 ± 12009.5; 20 mg/kg: 9901.7 ±5710 vs. model: 64333.8 ± 26943.7; *P*<0.05) were markedly reduced in the GM groups compared to that in controls (Figure 8A,B) Furthermore, the GM‐treated mice exhibited a significantly higher average body weight at week 2 than that of the control and TMZ groups (Figure S4A). Organ index confirmed the absence of significant treatment‐related toxicity (Figure S4B). The results showed that, similar to the TMZ treatment group, both 10 mg/kg and 20 mg/kg of GM treatment could significantly inhibit the growth of 4T1 tumor cells in the brain tissue (Figure 8C). Analysis of magnetically isolated primary AS demonstrated a dysregulation of the STAT3 pathway in the BrM model, evidenced by significantly upregulated p‐STAT3 and downregulated PIAS3. Notably, GM treatment concentration‐dependently decreased p‐STAT3 expression and significantly increased PIAS3 levels in AS (Figure 8D,E). Flow cytometry analysis of brain tissue revealed a significant decrease in immunosuppressive myeloid populations within the GM groups compared to that in controls: P‐MDSCs (5 mg/kg: 13.03% ± 0.21%; 10 mg/kg: 12.13% ± 0.42%; 20 mg/kg: 8.80% ± 0.28% vs. model: 14.23% ± 0.47%) and M‐MDSCs (5 mg/kg: 7.22% ± 0.56%; 10 mg/kg: 3.88% ± 0.21%; 20 mg/kg: 3.81% ± 0.20% vs. model: 7.26% ± 0.55%) (Figure 8F,G). Collectively, these results demonstrate that GM potently inhibits breast cancer brain metastasis in 4T1 combined with the 4T1 S100A14^OE^ EVs model, which is associated with the modulation of tumor‐promoting immune cells and a favorable safety profile.

To assess the broad‐spectrum potential of GM, we validated its efficacy in the lung cancer BrM model. Consistent with the 4T1 model, GM administration for 3 weeks significantly attenuated the metastatic burden in LLC model. Quantitative analysis demonstrated a profound reduction in whole‐body BLI signal intensity in the GM group (5 mg/kg: 473527.9 ± 256593.2; 10 mg/kg: 281555.8 ± 98252.7; 20 mg/kg: 100381.8 ± 67036.9) compared to that in the TMZ (310777.8 ± 119009.3) and model group (656749.3 ± 192404.1; Figure 8H). This significant suppression of BrM by GM was further confirmed by ex vivo brain BLI (Figure 8I). The results also showed that 10 mg/kg and 20 mg/kg of GM treatment could significantly inhibit the growth of LLC tumor cells in the brain tissue (Figure 8J). Western blot also demonstrated GM treatment concentration‐dependently decreased p‐STAT3 expression and significantly increased PIAS3 levels in AS (Figure 8K,L). Flow cytometry analysis of brain tissue also revealed significant decrease in immunosuppressive myeloid populations within the GM group compared to that in controls: P‐MDSCs (5 mg/kg: 9.09% ± 0.25%; 10 mg/kg: 8.93% ± 0.52%; 20 mg/kg: 8.91% ± 0.50% vs. model: 13.33% ± 0.40%) and M‐MDSCs (5 mg/kg: 6.54% ± 0.43%; 10 mg/kg: 4.44% ± 0.37%; 20 mg/kg: 5.12% ± 1.23% vs. model: 7.64% ± 0.60%) (Figure 8M,N). These findings verified that GM has extensive efficacy in treating BrM from different primary tumors (breast cancer and lung cancer), and underscore its therapeutic potential with minimal adverse effects.

### 2.7 GM Effectively Disrupted the S100A14‐PIAS3 Interaction, Remodeling the Immunosuppressive Microenvironment, and Inhibiting BrM

**FIGURE 9:**
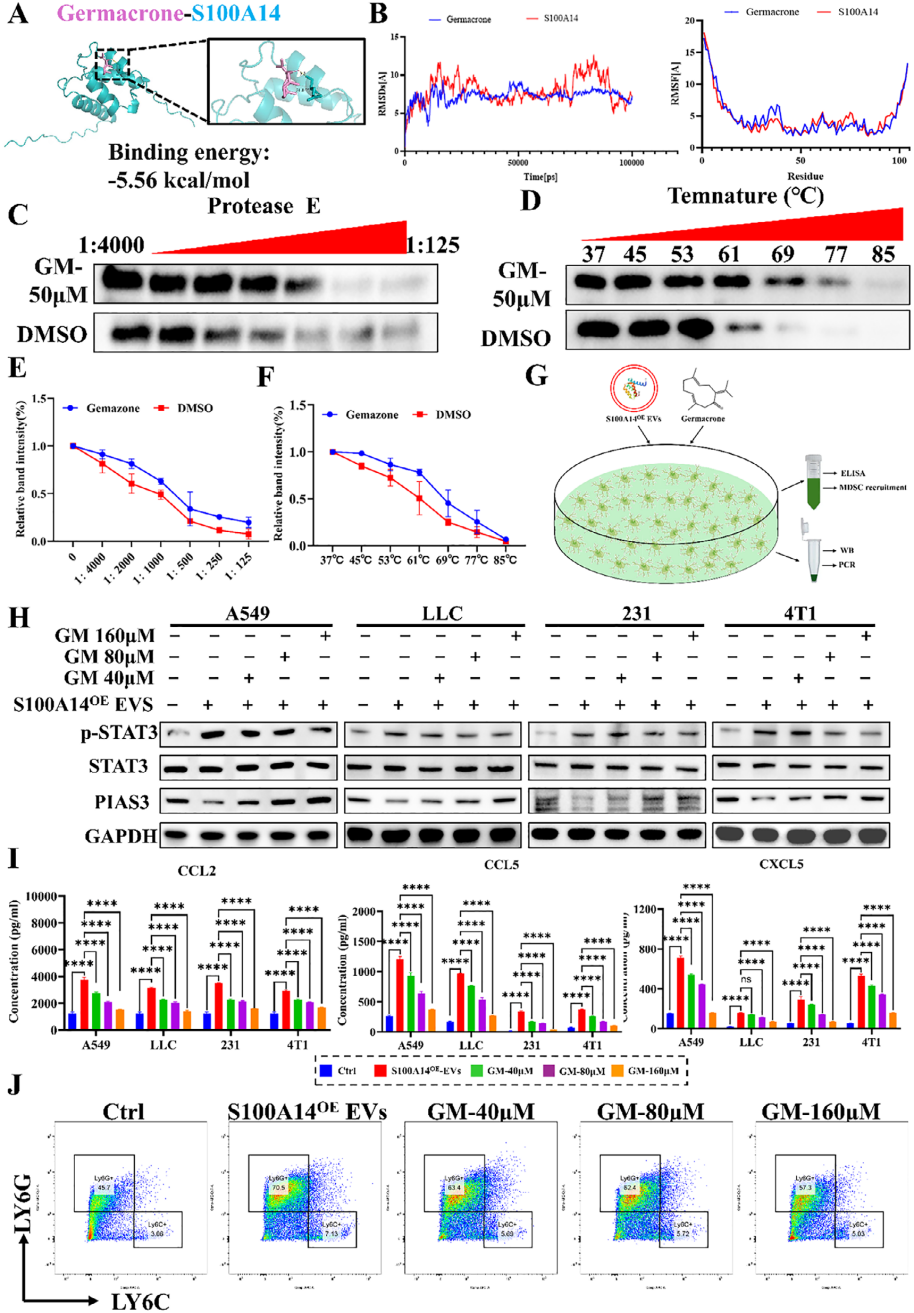
GM effectively disrupted the S100A14‐PIAS3 interaction, remodeling the immunosuppressive microenvironment and inhibiting BrM. (A) The 3D diagrams of GM and S100A14 molecular docking results. (B) The molecular dynamics simulations of S100A14 and GM. (C) The DARTS results of S100A14 and GM. (D) The CETSA results of S100A14 and GM. (E) The thermal stability of S100A14 under different concentrations of protease in presence of compound GM. (F) The thermal stability of S100A14 under different temperatures in presence of compound GM. (G) Schematic diagram of GM co‐cultured with S100A14^OE^ EVs in primary AS. (H) Protein expression levels of p‐STAT3 and STAT3 in primary AS co‐cultured with GM and S100A14^OE^ EVs. (I) ELISA results for CCL2, CCL5, and CXCL5 in the supernatant of primary AS co‐cultured with GM and S100A14^OE^ EVs. (J) Flow cytometry analysis of P‐MDSCs and M‐MDSCs in control, S100A14^OE^ EVs, and different concentrations of GM treatment groups. Data represent mean ± SEM. Significant difference versus control group, ^*^ *P*<0.05, ^**^ *P*<0.01, and ^***^ *P*<0.001.

We performed molecular docking analysis between GM and S100A14. The docking score of GM with S100A14 was ‐5.56 kcal mol^−1^, with the hydrogen bonds in GLU‐65. These results suggested that GM selectively binds to S100A14 (Figure 9A). MD simulation exhibited that the RMSD values of the complex system reached a stable state after 5 ns of simulation, and the RMSF curve exhibited very high values (around 15 Å) at the N‐terminal and C‐terminal regions, indicating that GM binding confers high stability to the S100A14 protein (Figure 9B). The DARTS assay revealed that the binding of GM to S100A14 stabilizes the conformation of the protein, thereby reducing its susceptibility to proteolytic degradation (Figure 9C). CETSA demonstrated that at higher temperatures (37–85°C) where S100A14 was largely denatured and degraded in the DMSO group, a significant fraction of the protein remained soluble and detectable in the GM‐treated groups (Figure 9D). This rightward shift in the thermal denaturation profile of the protein provided direct evidence that GM binds to S100A14 in a complex cellular environment (Figure 9E,F).

The EVs‐AS co‐culture model showed that GM potently inhibited S100A14^OE^ EV‐driven STAT3 pathway activation (Figure 9H). ELISA results showed that GM at the concentration of 160 µm could significantly decrease the secretion of pro‐inflammatory chemokines CCL2 (3739.13 ± 200.36 compared to 1511.83 ± 31.63 in A549; 3109.16 ± 67.47 compared to 1394.15 ± 84.02 in LLC; 3486.22 ± 19.64 compared to 1605.29 ± 27.47 in MDA‐MB‐231; 2907.71 ± 84.52 compared to 1678.57 ± 26.41 in 4T1), CCL5 (1200.06 ± 50.55 compared to 367.46 ± 4.84 in A549; 966.73 ± 7.99 compared to 267.46 ± 4.84 in LLC; 332.12 ± 16.2 compared to 37.84 ± 1.12 in MDA‐MB‐231; 366.74 ± 7.99 compared to 105.12 ± 4.66 in 4T1), and CXCL5 (715.30 ± 14.55 compared to 159.94 ± 2.36 in A549; 157.37 ± 6.50 compared to 72.51 ± 3.47 in LLC; 294.37 ± 27.50 compared to 73.21 ± 3.01 in MDA‐MB‐231; 531.69 ± 15.93 compared to 159.94 ± 2.36 in 4T1) in primary AS cells (Figure 9I). MDSCs recruitment experiments verified that GM significantly inhibited the migration of both P‐MDSCs (40 µM: 65.23%±3.44%; 80 µM: 62.1%±0.36%; 160 µM: 57.43% ± 0.49% compared to 69.9% ± 0.66%) and M‐MDSCs (40 µM: 6.13%±0.42%; 80 µM: 5.80%±0.17%; 160 µM: 5.14% ± 0.10% compared to 7.13% ± 0.05%) (Figure 9J). Collectively, these data established that GM directly targets S100A14, disrupting its downstream PIAS3‐STAT3 signaling cascade and thereby inhibiting astrocyte activation, which is pivotal for brain metastatic progression.

## 3 Discussion

Our study provides compelling evidence that EVs derived from BrM tumor cells play a pivotal role in establishing a permissive microenvironment for BrM. In vivo ICT BrM model confirmed that BM‐EVs not only significantly enhanced brain metastatic efficiency in both A549 LC (57.14% vs. 100%) and 4T1 breast cancer (50.00% vs. 83.33%), but also increased BLI in the body (A549:12787.2 ± 12950.94 vs. 188367.96 ± 183829.29; 4T1:17266.60 ± 24586.54 vs. 88932.20 ± 49807.12) and brain (391.81 ± 339.05 vs. 1217.29 ± 894.20; 4T1:264.46 ± 275.12 vs. 891.10 ± 571.47) (Figure 1E–G). DIA‐based quantitative proteomics of EVs from both cell lines and clinical samples revealed five proteins with consistently elevated levels in BM‐EVs, with S100A14 emerging as the most promising candidate. Mass spectrometry and western blot validation confirmed the significant upregulation of S100A14 in A549‐BM, 4T1‐BM, and LC‐BM EVs (Figure 2). S100A14^OE^‑EVs ICT mice models also further confirmed that S100A14^OE^ EVs markedly increased the incidence of BrM in A549 (83.33% vs. 33.33%), LLC (50.00% vs. 33.33%), 4T1 (88.89% vs. 44.45%), and MDA‐MB‐231 (66.67% vs. 33.33%) (Figure 3; Figure S2). This integrated multilevel analysis established S100A14 as a critical molecular mediator of BM EVs‐driven tumor BrM. Importantly, intersection analysis identifying S100A14 in clinical BrM patient‐derived EVs underscores its translational relevance as both a diagnostic biomarker and a potential therapeutic target.

The pathophysiology of BrM involves a complex interplay between circulating tumor cells and the brain microenvironment, in which AS plays a critical role [21, 23]. Increasing evidence has shown that RAs with elevated GFAP expression are key contributors to the pathogenesis of BrM [22]. Our results also confirmed that both BM‐EVs (Figure 1H) and S100A14^OE^‑EVs (Figure 3B,F; Figure S2E,F) induced profound microenvironmental changes characterized by enrichment of GFAP^+^ RAs surrounding metastatic lesions and robust recruitment of immunosuppressive MDSCs, including both P‐MDSCs (3.81% ± 0.23% vs. 9.57% ± 0.27% in 4T1 BM‐EVs; 7.27% ± 0.61% vs 15.77% ± 0.61% in LLC S100A14^OE^‑EVs; 10.30% ± 0.63% vs 18.95% ± 0.17% in 4T1 S100A14^OE^‑EVs) and M‐MDSCs (3.12% ± 0.11% vs. 6.71% ± 0.18% in 4T1 BM‐EVs; 2.29% ± 0.33% vs 4.92% ± 0.14% in LLC S100A14^OE^‑EVs; 4.56% ± 0.11% vs 5.70% ± 0.16% in 4T1 S100A14^OE^‑EVs) subsets (Figure 3C,D,F–H). The presence of this mechanism across different cancer types suggests that S100A14‐enriched EVs represent a universal pathway for metastatic progression through microenvironmental reprogramming via GFAP^+^‐activated AS enrichment and MDSCs recruitment. These findings suggest that S100A14 is a central regulator of the crosstalk between metastatic tumor cells and the brain microenvironment, offering new insights into the molecular mechanisms governing metastatic niche formation and suggesting novel strategies for targeting the S100A14 pathway to inhibit BrM progression.

Our study elucidated a novel mechanism by which S100A14^OE^ EVs reprogram RAs to create an immunosuppressive microenvironment conducive to BrM. We first demonstrated that S100A14^OE^ EVs efficiently crossed the BBB and were preferentially taken up by GFAP^+^AS in the peritumoral region (Figure 4A–D). Proteomic analysis revealed that S100A14^OE^ EVs treatment significantly upregulated 1179 proteins in the AS group, with enrichment in pathways critical for cell adhesion, junction assembly, and JAK‐STAT signaling (Figure 4G–I). Crucially, we observed sustained activation of STAT3 signaling in AS, as evidenced by increased phosphorylated STAT3 levels (Figure 4J). This activation triggered the secretion of key pro‐inflammatory chemokines, including CCL2, CCL5, CXCL5, CXCL10, and IL‐6, as validated by Luminex assay and qRT‐PCR (Figure 5A–C). The importance of these chemokines was underscored by their elevated levels in the cerebrospinal fluid of mice with BrM and their strong correlation with metastatic burden (Figure 5D,E). Functional assays confirmed that conditioned media from S100A14^OE^ EV‐treated AS robustly recruited MDSCs, particularly P‐ and M‐MDSCs, via chemokine‐dependent mechanisms (Figure 5G,H). These findings established that S100A14^OE^ EVs exploit astrocytic STAT3 signaling to secrete chemokines that recruit immunosuppressive MDSCs, thereby fostering a permissive niche for BrM.

Mechanistically, we identified PIAS3 as the direct molecular target of S100A14. Through integrated structural and biochemical approaches, including molecular docking (Figure 6A), Y2H assay (Figure 6B), CO‐IP (Figure 6C), and BIFC (Figure 6D), we demonstrated direct binding between S100A14 and PIAS3. Most importantly, the SPR assay confirmed that fast association and dissociation steps were observed upon S100A14 injections on the aptamer surface. However, nonconventional SPR signals were observed (Figure 6E). The reference channel shows no signal, indicating that the S100A14 protein does not bind to the matrix (Figure S3F left). Meanwhile, the value of the active channel is negative (Figure S3F right), which suggests that the conformation transition of the PIAS3 protein after the two proteins combine as previously reported for proteins [42, 43, 44, 45]. This interaction was dependent on residue 154 of PIAS3 and occurred primarily in the extranuclear compartment of AS (Figure 6C–E). Functionally, this binding antagonized PIAS3's native role as a suppressor of STAT3 transcriptional activity. Knockdown of PIAS3 in AS phenocopied the effects of S100A14^OE^ EVs treatment, leading to STAT3 phosphorylation and subsequent chemokine secretion (CCL2, CCL5, and CXCL5) (Figure 7A–G). The S100A14‐PIAS3‐STAT3 axis represents a previously unrecognized pathway in tumor‐induced astrocytic reprogramming.

Importantly, we are the first to identify GM as a natural compound that disrupts the S100A14‐PIAS3‐STAT3 axis by targeting S100A14, thereby inhibiting STAT3 activation and chemokine‐driven MDSC recruitment. Our in vivo investigation revealed the remarkable efficacy of GM in suppressing metastatic progression in both lung and breast cancer models. In the LLC BrM model, GM treatment resulted in a profound reduction of metastatic burden, with in vivo BLI decreasing from 656749.3 ± 192404.1 in control to 100381.8 ± 67036.9 in 20 mg/kg GM‐treated groups, representing approximately 6.5‐fold reduction. This robust anti‐metastatic activity was consistently observed in 4T1 breast cancer model, where GM significantly attenuated both in vivo (711853.3 ± 593256.9 vs. Control: 18801816.0 ± 5750980.1) and brain‐specific (9901.7 ± 5710 vs. Control: 64333.8 ± 26943.7) metastatic signals. Notably, GM‐treated mice maintained a stable body weight and showed no significant organ toxicity, suggesting superior tolerability compared to that of conventional TMZ treatment. The therapeutic efficacy was further associated with significant modulation of the tumor microenvironment, particularly through reduction of immunosuppressive myeloid populations including both P‐MDSCs (13.3% ± 0.40% vs. 8.93% ± 0.52% in LLC; 14.23% ± 0.47% vs. 8.80% ± 0.28% in 4T1) and M‐MDSCs (7.64% ± 0.60% vs. 4.44% ± 0.37% in LLC; 7.26% ± 0.55% vs. 3.81% ± 0.20% in 4T1) in the brain tissue. These findings suggest that GM is a promising therapeutic agent with efficacy and safety advantages over existing treatments.

Beyond demonstrating the therapeutic efficacy, we also elucidated the precise underlying molecular mechanism of GM. Using integrated computational and experimental approaches, we established that GM directly targets S100A14 with exceptional affinity and specificity. Molecular docking revealed high‐affinity binding (docking score: −5.56 kcal mol^−^^1^) with specific interactions involving key residues like GLU‐65. While DARTS and CETSA provided orthogonal validation of direct target engagement. Functionally, GM potently inhibited S100A14^OE^ EV‐driven STAT3 pathway activation in AS, attenuating both the protein and mRNA expression of key STAT3 pathway components. The convergence of structural, biophysical, and functional evidence establishes GM as a lead compound that specifically targets the molecular cascade driving astrocyte‐mediated immunosuppressive niche formation, thus offering a novel therapeutic strategy for preventing BrM through microenvironment modulation.

In summary, to the best of our knowledge, our study is the first to reveal a novel mechanism by which tumor‐derived S100A14 EVs promote tumor BrM, wherein tumor‐derived S100A14 EVs directly target PIAS3 to reprogram AS, thereby recruiting MDSCs and creating an immunosuppressive microenvironment. Furthermore, we identified the natural compound, GM, as a promising therapeutic agent that effectively inhibits the binding of S100A14 and PIAS3, suppresses the secretion of pro‐inflammatory chemokines by AS, and reprograms the brain tumor immune landscape to prevent BrM.

## 4 Materials and Methods

### 4.1 Cell Culture

#### 4.1.1 Cell Culture and Compounds

The human LC cell line A549‐luc was purchased from Shanghai Hengrui Biotechnology Co., Ltd. and cultured in RPMI‐1640 medium containing 10% FBS and 1% penicillin‐streptomycin. The LLC‐luc mouse LC cell line was obtained from Guangzhou Kain Biotechnology Co., Ltd. and grown in Dulbecco's modified DMEM medium (DMEM) with 10% fetal bovine serum (FBS) and 1% penicillin‐streptomycin. The human breast cancer cell line, MDA‐MB‐231‐luc, was established by Shanghai Oubi Biotechnology Co., Ltd. and cultured in L15 medium containing 10% FBS and 1% penicillin‐streptomycin. The 4T1‐luc mouse breast cancer cell line was procured from Beijing Bokai Biotechnology Co., Ltd. and maintained in DMEM with 10% FBS and 1% penicillin‐streptomycin. MDA‐MB‐231‐luc cells are required to be maintained in a 37°C incubator with 95% humidity and 0% CO_2_, while all other cells are cultured in a 37°C incubator with over 95% humidity and 5% CO_2_. Temozolomide (TMZ) was purchased from Sigma‐Aldrich. GM was purchased from Chengdu Mansite Biotechnology Co., Ltd. Anti‐mouse CCL2 (A2132, Selleck), CCL5 (MA5‐23839, Thermo Fisher Scientific), and CXCL5 (BE0449, Thermo Fisher Scientific) antibodies were used to validate the in vitro MDSCs recruitment experiments.

#### 4.1.2 Primary Immune Cells Isolation

The whole mouse brain was rapidly extracted and mechanically dissociated into a single‐cell suspension using a 1 mL syringe plunger. The tissue homogenate was filtered through a 70‐µm cell strainer with RPMI‐1640. The filtrate was centrifuged at 2000 rpm for 10 min, and the supernatant was discarded. The cell pellet was resuspended in 10 mL of 30% Percoll and carefully layered onto a pre‐prepared 3 mL density cushion of 70% Percoll in a 15 mL centrifuge tube. This discontinuous gradient was centrifuged at 1500 rpm for 40 min with the brake disabled. The intermediate layer, containing the cells of interest, was aspirated and transferred to a new tube. Finally, the cells were washed twice with PBS by centrifugation at 2000 rpm for 10 min. Freshly isolated brain immune cells were stained for CD45 (30‐F11, FITC, 103108, BioLegend), CD11b (M1/70 PE, 101208, BioLegend), Ly6C (HK1.4 APC, 17‐5932‐82, Invitrogen), or Ly6G (1A8, APC‐Cy7, 127624, BioLegend) on ice for 30 min in the dark. Cells were filtered through a 35‐µm strainer into FACS tubes with 300 µL FACS buffer and detected by a flow cytometer (BD FACSAriaII).

#### 4.1.3 Primary AS Isolation and Transient Transfection

The procedure of primary AS isolation from newborn mice was as follows: brain tissues were dissected under sterile conditions, and the meninges, associated vasculature, olfactory bulbs, cerebellum, midbrain, and hippocampi were excised. The isolated cortices were minced into approximately 1 mm^3^ pieces and enzymatically digested in Hank's Balanced Salt Solution (HBSS) containing 0.25% EDTA‐trypsin for 30 min at 37°C. Digestion was terminated by adding DMEM supplemented with 10% FBS, sequentially filtering through a 70‐µm cell strainer, and centrifuging at 1500 rpm for 10 min. The cell pellet was resuspended in DMEM supplemented with 10% FBS and 1% penicillin‐streptomycin and seeded into culture flasks pre‐coated with 0.01% poly‐d‐lysine (PDL). Cells were cultured in a humidified incubator at 37°C with 5% CO_2,_ and medium was replaced every 2–3 days. High‐purity AS were obtained by shaking at 200 rpm overnight and confirmed by GFAP immunofluorescence (IF) staining.

Primary AS isolated from tumor‐bearing mice were sorted using magnetic‐activated cell sorting (MACS) with anti‐ACSA‐2 MicroBeads (130‐097‐678, Miltenyi Biotec) following the manufacturer's instructions. In brief, single‐cell suspensions were prepared by mechanical homogenization and sequential filtration through 70 and 40‐µm strainers. After FcR blocking, the cells were labeled with ACSA‐2 MicroBeads and passed through an LS Column in a MiniMACS Separator. Magnetically retained cells were eluted as purified AS. Cell purity was assessed using flow cytometry (ACSA‐2‐PE) and IF staining for GFAP.

To knock down PIAS3 in primary AS cells, they were seeded at 3 × 10^6^ cells per well in 6‐well plates. After 24 h, the cells were transfected using Lipofectamine 3000 (L3000015; Thermo Fisher Scientific). For each well, a complex was formed by mixing two solutions: 1) 3.75 µL Lipofectamine 3000 in 125 µL Opti‐MEM, and 2) 2.5 µg sh‐pias3 plasmid with 5 µL P3000 Enhancer in 125 µL Opti‐MEM. The combined mixture was incubated for 15 min at room temperature before application to the cells. Transfected cells were incubated for 48 h before harvesting for analysis.

#### 4.1.4 Co‐Culture of AS and EVs

An in vitro coculture model was established to simulate the interactions between tumor‐derived EVs and primary AS. Primary AS were cultured until 80% confluence and then treated with complete medium containing tumor‐derived EVs at a final concentration of 20 µg/mL for 6 h. After treatment, both the conditioned medium and AS were collected for further analysis.

#### 4.1.5 MDSCs Recruitment and Flow Cytometry

An ex vivo chemotaxis assay was performed to evaluate the effects of chemokines on MDSC recruitment in AS coculture‐conditioned medium. Bone marrow cells from tumor‐bearing mice were aseptically isolated from their tibiae and femora. Briefly, the ends of the bones were cut open with ophthalmic scissors, and the marrow was flushed into a 50 mL tube using PBS with a 1 mL syringe until the bones turned white. The cell suspension was then centrifuged at 800 rpm for 5 min. The erythrocytes were lysed by resuspending the pellet in 5 mL of RBC lysis buffer for 5 min at room temperature, followed by centrifugation. The resulting leukocytes were washed twice with PBS before use.

For the chemotaxis assay, primary bone marrow cells were seeded in 6‐well Transwell plates (14122, LABSELECT) in the upper chamber, and conditioned medium was added in the lower chamber. To assess the role of specific chemokines, neutralizing antibodies against CCL2 (A2132, Selleckchem), CCL5 (BE0449; BioXcell), and CXCL5 (MA5‐23839; Thermo Fisher Scientific) were added to the lower chambers. After a 4‐h migration period, cells in the lower chamber were harvested, stained with a multicolor antibody panel (CD45‐FITC, CD11b‐PE, Ly6C‐APC, and Ly6G‐APC‐Cy7), and analyzed using flow cytometry to quantify the migrated MDSCs.

### 4.2 Exosomes (EVs)

#### 4.2.1 EVs Extraction

The experimental steps for isolating EVs from cells were as follows: cells were cultured in serum‐free medium for 48 h. The conditioned medium was collected and subjected to a series of centrifugation steps: 1500 rpm for 10 min to remove intact cells; 8000 rpm for 30 min to pellet cellular debris; and 440000 rpm for 90 min at 4°C to pellet the EVs. The EVs pellet was washed with PBS, followed by a second ultracentrifugation step under the same conditions (440000 rpm, 90 min). The final EVs pellet was resuspended in a suitable volume of PBS and stored at −80°C for subsequent experiments.

Plasma‐derived EVs were isolated by size exclusion chromatography (SEC) using a qEV column (Izon Science, Oxford, UK), according to the manufacturer's protocol. Briefly, the column was equilibrated with 10 mL of PBS. Subsequently, plasma (0.5 mL) was loaded onto the column, and elution was performed with particle‐free PBS. Fractions 7–9, which typically contained the highest concentrations of purified EVs, were collected and pooled. The pooled EV‐containing fractions were stored at −80°C for subsequent analysis [46, 47].

#### 4.2.2 Electron Microscopy Analysis of EVs

The morphology and structural integrity of the isolated EVs were characterized using transmission electron microscopy (TEM; Wuhan Sevier Biotechnology Co., Ltd). Briefly, EVs samples were negatively stained with uranyl acetate on a formvar‐carbon‐coated copper grid and air‐dried. The EV morphology was examined using a Hitachi HT7800 TEM microscope operating at 100 kV.

#### 4.2.3 Nanoparticle Tracking Analysis of EVs

The size and concentration of EVs were analyzed using Nanoparticle Tracking Analysis (NTA) conducted by Wuhan Sevier Biotechnology Co., Ltd. Briefly, the isolated EVs fraction was diluted in PBS. Analysis was performed using a nanoparticle‐tracking analyzer (ZetaView, Particle Metrix). For each sample, three 30‐s videos were recorded, and the data were processed using NTA software to determine the particle size distribution profile and concentration.

#### 4.2.4 Western Blot Analysis of EVs

Protein expression in the isolated EVs was characterized using western blot analysis. EVs were lysed in RIPA buffer, and the protein concentration was quantified. Proteins were separated by sodium dodecyl sulfate‐polyacrylamide gel electrophoresis (SDS‐PAGE) and transferred to PVDF membranes. After blocking, the membrane was probed with primary antibodies against EVs markers such as CD81 (52892T, CST), Alix (18269T, CST), TSG101 (72312T, CST), and HSP70 (46477T, CST). Following incubation with a horseradish peroxidase‐conjugated secondary antibody, protein bands were detected using an enhanced chemiluminescence imaging system.

#### 4.2.5 Fluorescent Labeling of EVs

To achieve visualization of EVs, two lipophilic membrane dyes were employed for distinct applications: DiR (CF0304, CYTOCH) for in vivo near‐infrared imaging and DiL (C1036, Beyotime) for in vitro co‐localization studies. Briefly, the isolated EV pellet was resuspended in PBS and incubated with the respective dyes at a final concentration of 10 µm for 20 min at 37°C in the dark. The labeling reaction was stopped by adding a 10‐fold volume of cold PBS. To remove the unincorporated dye and ensure signal specificity, the labeled EVs were subsequently re‐isolated via ultracentrifugation. The final labeled EV pellet was resuspended in PBS for further use.

### 4.3 Animals

#### 4.3.1 Intracardiac BrM Model

The intracardiac injection model (ICT) [48] was used to explore BrM of the breast and LC, according to our previous study. Specifically, 1 × 10^5^ 4T1‐luc, 2 × 10^6^ LLC‐luc, 1.5 × 10^6^ 231‐luc, and 5 × 10^5^ A549‐luc cells were injected into the left ventricle of 4 to 6‐week‐old female BALB/c, C57BL/6, or BALB/c‐nude mice using a 26G needle. The injected mice were weighed every other day, and neurological symptoms were monitored. EVs were injected via the tail vein three times per week at a dose of 20 µg per mouse. All mice were purchased from the Guangdong Medical Experimental Animal Center, and all animal experiments were approved by the Animal Care and Use Committee of Guangzhou University of Chinese Medicine (SYXK‐2024‐0144 Animal Use License). BrM of the mice was detected by in vivo bioluminescence imaging. Briefly, mice were intraperitoneally injected with 100 µL 1.5% D‐luciferin (Biosharp, Anhui, China) and placed in the Xenogen IVIS system (IVIS Lumina XRMS, Series III) for in vivo imaging detection. Tumor‐bearing mice were imaged every 7 days to monitor bioluminescence intensity (BLI) and distribution.

#### 4.3.2 Intracarotid BrM Model and Drug Treatment

The intracarotid injection model (ICD) [48] was used to evaluate the inhibitory effects of curdione on BrMs of the breast and LC, according to our previous study. Specifically, 3 × 10^3^ 4T1‐luc cells or 2 × 10^6^ LLC‐luc cells were injected into the carotid arteries of 4‐week‐old female BALB/c or C57BL/6 mice using a 31G needle. The injected mice were weighed every other day, and neurological symptoms were monitored. EVs were injected via the tail vein [14, 49, 50] three times per week [51] at a dose of 20 µg [52] per mouse. Three days later, the mice were divided into three groups and treated with TMZ (25 mg/kg) or GM (5, 10, or 20 mg/kg) for 2 weeks. The in vivo bioluminescence intensity of each mouse was monitored, and the mice were sacrificed when their body weight was less than 15 g. Following dissection, the bioluminescence intensity of the brain tissue was measured in situ. Subsequently, primary cells were isolated from fresh brain tissue for flow cytometry.

### 4.4 Clinical Samples

Serum samples from five clinical patients with LC and five patients with LC BrM were collected. All experiments were approved by the Clinical Ethics Committee of the Second Affiliated Hospital of Guangzhou University of Chinese Medicine (approval no. ZE2025‐005). All patients signed an informed consent form, and detailed patient information is shown in Table 1.

**TABLE 1:** Information of clinical patient samples.

No.	Gender	Age	Types of Lung Cancer	Staging of Cancer
1	male	55	Lung adenocarcinoma	T1CN0M0 IA3
2	female	57	Lung adenocarcinoma	T1cN0M0 IA2
3	female	69	Lung adenocarcinoma	T1cN0M0 IA2
4	female	53	Lung adenocarcinoma	T1BN0M0 IA2
5	female	81	Lung adenocarcinoma	T1BN0M0 IA2
1	female	69	Lung carcinoma	Brain metastasis
2	male	68	Lung carcinoma	Brain metastasis
3	male	85	Lung carcinoma	Brain metastasis
4	female	68	Lung carcinoma	Brain metastasis
5	female	59	Lung carcinoma	Brain metastasis

### 4.5 DIA‐Based Quantitative Proteomic Analysis

Data independent acquisition (DIA) quantitative proteomics was used to explore the key target proteins and signaling pathways involved in LC‐BM and to systematically identify them in human plasma EVs. First, the EVs from the LC and BM groups were purified using qEV columns and enriched with magnetic beads. Second, after lysis of 30 µg protein solution to dissolve the precipitate, trypsin was added for overnight digestion. Third, the digested peptide samples were analyzed using nano‐LC (Easy nLC1200, Thermo Fisher Scientific) coupled with an Orbitrap Exploris 480 FAIMS Pro mass spectrometer (Thermo Fisher Scientific). Raw files were processed in Spectronaut (version 15.0; Biognosys AG 2013), and database search was performed against UniProt. Mass spectrometry and database analyses were performed by Shanghai Huaying Biomedical Technology Co., Ltd.

DIA quantitative proteomics was used to explore the key target proteins and signaling pathways involved in the exosome‐mediated activation of primary AS. First, the cells were collected 6 h post‐co‐culture; proteins were then extracted, digested, and desalted using desalting columns. After freezing and concentration, the samples were separated on an ES906 C18 analytical column (5010‐21701, GL Science). The liquid chromatography separation output was directly connected to an Orbitrap Astral platform for mass spectrometry (Thermo Fisher Scientific). The separated peptides were ionized using an electrospray ionization (ESI) ion source and analyzed directly using DIA in a mass spectrometer. Mass spectrometry and database analyses were performed by Shanghai Huaying Biomedical Technology Co., Ltd.

### 4.6 Molecular Docking Analysis

Protein–protein docking analysis was performed to predict the interaction between S100A14 and PIAS3. The 3D structures of PIAS3 (X‐ray crystal structure, PDB: 4MVT) and S100A14 (NMR solution structure, PDB: 2M0R) were retrieved from the Protein Data Bank. The HDOCK server, which utilizes a hybrid algorithm of template‐based and ab initio‐free docking, was used for rigid‐body docking. The resulting docking complexes were analyzed to evaluate binding affinity using the PDBePISA database for interface analysis. Specific residues involved in the putative binding sites were visualized and analyzed using the PyMOL Molecular Graphics System.

A molecular docking simulation was conducted to investigate the potential binding of GM to S100A14. The 3D chemical structure of GM was obtained from the PubChem database. The protein structure of S100A14 was prepared using PDB entry 2M0R. Docking simulations were performed using the AutoDock software, which explores the binding conformation and affinity of the ligand within the binding pocket of the protein.

### 4.7 Yeast Two‐Hybrid (Y2H) Assay

Protein‐protein interactions between PIAS3 and S100A14 were investigated using the Y2H system. The coding sequences for PIAS3 and S100A14 were cloned into bait (pGBKT7) and prey (pGADT7) vectors, and all recombinant plasmids were constructed by Wuhan JinKaiRui Biological Engineering Co., Ltd. Prior to the interaction test, the bait plasmid (pGBKT7‐PIAS3) was co‐transformed with an empty prey vector (pGADT7) in the Y2H Gold yeast strain to test for autoactivation. Transformed yeasts were plated on synthetic dropout media lacking Trp and Leu (DDO), as well as on more stringent selection media lacking Trp, Leu, and His (TDO) or Trp, Leu, His, and Ade (QDO). The absence of growth on TDO and QDO media confirmed that the bait protein did not autonomously activate the reporter genes. For the interaction assay, validated bait and prey plasmids (pGADT7‐S100A14) were co‐transformed into yeast. Protein interactions were subsequently confirmed by growth on stringent QDO medium and further validated using a serial dilution spot assay.

### 4.8 Co‐Immunoprecipitation (CO‐IP) Assay

Protein‐protein interactions between S100A14 and PIAS3 were validated by co‐immunoprecipitation (Co‐IP) using a commercial kit (P2179S; Beyotime) and a magnetic rack. Briefly, cell lysates were incubated overnight at 4°C with primary antibodies against PIAS3 (sc‐46682; Santa Cruz Biotechnology) or a control rabbit IgG (7074; Cell Signaling Technology). Protein A/G magnetic beads were then added to capture the antibody‐antigen complexes. After extensive washing, the bound proteins were eluted and subjected to western blot analysis using an anti‐S100A14 antibody (10489‐1‐AP; Servicebio). For binding site mapping, a FLAG‐tagged PIAS3 mutant (R154A) was generated via site‐directed mutagenesis by Changsha Zeqiong Biological Technology Co. Ltd. The mutant protein was pulled down using an anti‐FLAG antibody, and co‐precipitation of S100A14 was analyzed by western blotting.

### 4.9 Bimolecular Fluorescence Complementation Assay (BIFC)

The protein‐protein interaction between PIAS3 and S100A14 was visualized in live cells using a bimolecular fluorescence complementation (BIFC) assay. The coding sequences of PIAS3 and S100A14 were cloned into the pcDNA3.1‐YNE and pcDNA3.1‐YCE vectors, which encode the N‐ and C‐terminal fragments of the Venus fluorescent protein, respectively. For the assay, cells were seeded on glass coverslips in a 24‐well plate and grown to 60–70% confluence. The cells were then co‐transfected with the PIAS3‐YNE and S100A14‐YCE plasmid pairs using Lipofectamine 3000 reagent according to the manufacturer's instructions. Briefly, a total of 2 µg of plasmid DNA was mixed with the transfection reagent in serum‐free DMEM, incubated for 15 min to form complexes, and then applied to the cells. After 4–6 h of incubation at 37°C, the transfection mixture was replaced with complete culture medium, and the cells were cultured for an additional 48 h to allow for protein expression and complementation. Subsequently, the cells were fixed with 4% paraformaldehyde for 15 min, washed thoroughly with PBS, and mounted onto glass slides using anti‐fade mounting medium. The reconstituted Venus fluorescence signal, indicative of a direct interaction between PIAS3 and S100A14, was captured using laser scanning confocal microscopy.

### 4.10 Surface Plasmon Resonance (SPR) Assay

The interaction between S100A14 protein with PIAS3 was monitored by SPR using a BIAcore T200 (Cytiva), carried out at 25°C in LMW multi‐cycle mode. Experiments were performed using Wayen Biotechnologies Inc. The CM5 biosensor chip (Cytiva) was immobilized with the PIAS3 protein according to the manufacturer's amine‐coupling chemistry protocol (Cytiva). The S100A14 used for this assay was in a buffer containing PBS. Various concentrations of S100A14 were then passed through the chip, and the real‐time response was recorded. The concentrations of S100A14 were 0, 31.25, 62.5, 125, 250, 500, and 1000 nm. The equilibrium dissociation constants (binding affinity, KD) for each pair of interactions were calculated using BIAcore T200 evaluation software (Cytiva).

### 4.11 Molecular Dynamics (MD) Simulation

MD simulations were performed to investigate the stability and interaction dynamics of the S100A14‐GM complex. The initial structure of S100A14 (PDB: 2M0R) was complexed with GM, the geometry of which was optimized at the HF/6‐31G* level. The system was solvated in a TIP3P water box with 10 Å padding and neutralized with 0.15 M NaCl. After energy minimization and stepwise equilibration in the NVT/NPT ensembles, a 200 ns production simulation was conducted at 310 K and 1 atm using the CHARMM36 force field in GROMACS. Trajectory analyses included root‐mean‐square deviation (RMSD) and root‐mean‐square fluctuation (RMSF) for stability, MM/PBSA for binding free energy, hydrogen bonding analysis, and principal component analysis for collective motions.

### 4.12 Cellular Thermal Shift (CETSA) Assay

S100A14^OE^ tumor cells were seeded into 75 cm^2^ flasks and grown to 70–80% confluence to assess the binding affinity of S100A14 for GM. Cells were treated with GM (50 µM) or solvent (DMSO) for 12 h. Cells were harvested and resuspended in 700 µL of PBS containing protease inhibitors and phosphorylation inhibitors. The cell suspension was aliquoted equally into seven PCR tubes (∼140 µL each). Samples were subjected to a defined temperature gradient (temperature range, 37°C, 45°C, 53°C, 61°C, 69°C, 77°C, and 85°C) for 5 min in a thermal cycler, followed by immediate cooling on ice for 3 min. Cells were lysed by sonication (three pulses of 10 s each at 30% amplitude on ice) in RIPA buffer containing inhibitors. Lysates were clarified by centrifugation (12,000 rpm at 4°C for 15 min). Supernatants were collected, mixed with 5 × Loading buffer, and denatured (95°C for 5 min). Protein samples were analyzed by western blotting.

### 4.13 Drug Affinity Responsive Target Stability (DARTS) Assay

A systematic drug‐protein interaction assay was developed using S100A14^OE^ tumor cells to investigate the binding interaction between S100A14 and GM. The cells were harvested, lysed, and quantified using the BCA assay. The clarified protein extract was divided equally into two aliquots and incubated with GM (50 µm) or solvent (DMSO) on ice for 1 h with continuous vortexing. Subsequently, samples were subjected to serial protease digestion with Pronase (concentration range: 1:4000, 1:2000, 1:1000, 1:500, 1:250, and 1:125) for 30 min at 37°C, followed by inactivation at 95°C for 10 min. Protein samples were analyzed by western blotting.

### 4.14 IF, Hematoxylin and Eosin Staining (H&E)

IF experiments were conducted according to the standard protocol of a multiplex fluorescent staining kit (AFIHC034; Aifang Biotechnology). Primary antibodies against p‐STAT3 (9145; CST), PIAS3 (sc‐46682; Santa Cruz Biotechnology), and GFAP (sc‐22673; Santa Cruz Biotechnology) were used. The secondary antibodies used were Alexa Fluor594‐labeled AffiniPure goat anti‐rabbit IgG (111‐585‐114; Jackson) and Alexa Fluor488‐labeled AffiniPure goat anti‐mouse IgG (115‐545‐003; Jackson). The cells were fixed in 4% polyformaldehyde solution on a cover slip for 20 min, followed by permeabilization with PBS containing 0.1% Triton X‐100 (0694, GBCBIO). Then, cells were blocked with goat serum for 20 min and then incubated overnight at 4°C with the primary antibodies. The next day, the cells were washed thrice with PBST, incubated with secondary antibodies for 1 h, and re‐stained with DAPI to quench the fluorescence. Cell fluorescence was detected using a TCS SP8 confocal microscope (Leica, Wetzlar, Germany). ImageJ software (Leica LAS X hardware configurator, Wetzlar, Germany) was used to analyze the number of positively stained cells and comprehensive optical density.

GFAP and S100A14 expression in the metastatic brain tissue was detected using IF. Metastatic brain tissues were fixed, embedded in paraffin, and sectioned. Paraffin‐embedded tumor sections were dewaxed and antigenically repaired to inactivate endogenous peroxidase activity, followed by incubation with bovine serum albumin. The sections were then incubated overnight with the primary antibody GFAP and S100A14 at 4°C, followed by incubation with the corresponding secondary antibody. Finally, cell nuclei were stained with an anti‐fluorescence quencher mounting medium containing DAPI.

H&E staining of the brain tissue was performed according to a standard procedure. The sections were first subjected to gradient dewaxing, followed by staining with hematoxylin for 30 s. After rinsing with water, the cells were performed using 0.5% hydrochloric acid–ethanol. The sections were counterstained with eosin for 2 s, rinsed under running water, dehydrated through a gradient ethanol series, and mounted with a neutral resin after drying. Images of brain tissue sections were obtained using a Leica microscope.

### 4.15 Quantitative Real‐Time PCR (qRT‐PCR) and Western Blot (WB)

Total RNA was extracted from the cells using TRIzol reagent (Thermo Fisher Scientific). The quality and quantity of total RNA samples were assessed using a NanoDrop spectrometer. Reverse transcription was performed using the SYBR Green Pro Taq HS kit (AG11718 AG). Quantitative real‐time PCR (qRT‐PCR) was conducted using the SYBR Green Pro Taq HS kit following the manufacturer's instructions. The primer sequences for CXCL10, CX3CL1, IL‐6, CCL22, CXCL16, CCL11, CXCL11, CCL5, CXCL5, and CCL2 are listed in Table 2. The data were collected and analyzed using QuantStudio Design & Analysis software, with β‐Actin used as the internal reference gene.

**TABLE 2:** Primers List.

Gene	Forward (5’‐3’)	Reverse (5’‐3’)
CXCL10	CCAAGTGCTGCCGTCATTTTC	GGCTCGCAGGGATGATTTCAA
CX3CL1	ACGAAATGCGAAATCATGTGC	CTGTGTCGTCTCCAGGACAA
IL‐6	GATGCAACCAAACTGGATATAATC	GAGCATTGGAAGTTGGGGTA
CCL22	AGGTCCCTATGGTGCCAATGT	CGGCAGGATTTTGAGGTCCA
CXCL16	CCTTGTCTCTTGCGTTCTTCC	TCCAAAGTACCCTGCGGTATC
CCL11	GAATCACCAACAACAGATGCAC	ATCCTGGACCCACTTCTTCTT
CXCL11	GGCTTCCTTATGTTCAAACAGGG	GCCGTTACTCGGGTAAATTACA
CCL5	ATATGGCTCGGACACCACTC	TTCGAGTGACAAACACGACTG
CXCL5	GCCCCTTCCTCAGTCATAGC	AGCTTTCTTTTTGTCACTGCCC
CCL2	CACTCACCTGCTGCTACTCA	TGAGCTTGGTGACAAAAACTACAG
β‐actin	CACTGTCGAGTCGCGTCC	TCATCCATGGCGAACTGGTG

Cells were lysed in RIPA buffer (G3424, GBCBIO) containing protease and phosphatase inhibitors (G2007, Servicebio), and total protein was extracted by centrifugation. The protein concentration was determined using a BSA protein assay kit (P0010, Beyotime). Approximately 20 µg of protein was loaded onto SDS‐PAGE for separation, followed by electrophoretic transfer to a PVDF membrane (Millipore, USA). The membrane was blocked and washed, and then incubated overnight with the following primary antibodies at 4°C: p‐STAT3 (9145, CST), PIAS3 (sc‐46682, Santa Cruz), STAT3 (F0200, Selleck), and GAPDH (60004‐1, Proteintech). The following day, the membranes were incubated with secondary antibody solutions containing anti‐rabbit IgG (7074, CST) and anti‐mouse IgG (7076, CST). Finally, the membranes were visualized using the ECL gel imaging system (Thermo Scientific Chemiluminescent Substrate, USA).

### 4.16 Statistical Analysis

Statistical analyses were performed using GraphPad Prism 8.2 software. All data are presented as mean ± standard error of the mean (SEM). Differences among multiple groups were analyzed using one‐way analysis of variance (ANOVA), and t‐tests were employed for pairwise comparisons. *P* <0.05 was considered statistically significant.

## Author Contributions

Rong‐Rong Zhang and Qian Feng analyzed the data, wrote the manuscript, and prepared the figures. Xia Yang and Xue‐Yu Wang designed and performed the experiments. Ming‐Rui Wang and Zhuo‐Hong Cai verified all the data and conducted statistical validation. Lin An and Cai‐Jun Xie collected the clinical samples and performed the experiments. Cai‐Yan Wang and Zhong‐Qiu Liu revised the manuscript. All the authors have read and approved the final manuscript.

## Conflicts of Interest

The authors declare no conflict of interest.

## Supporting information




**Supporting File**: advs75282‐sup‐0001‐SuppMat.docx.

## Data Availability

The data that support the findings of this study are available from the corresponding author upon reasonable request.
