# Supplementary material for: S100A14 in Tumor‐Derived EVs Targets PIAS3 to Reprogram Astrocytes and Induce Immunosuppressive Microenvironment Promoting Brain Metastasis and Germacrone Reversal Effect
Source: Adv Sci (Weinh). 2026 Apr 10;13(39):e22921. doi: 10.1002/advs.202522921 (PMC13334997; doi:10.1002/advs.202522921)
Supplement: Supplementary file 1 — Supporting File: advs75282‐sup‐0001‐SuppMat.docx. [file ADVS-13-e22921-s001.docx]

**Figure S1**


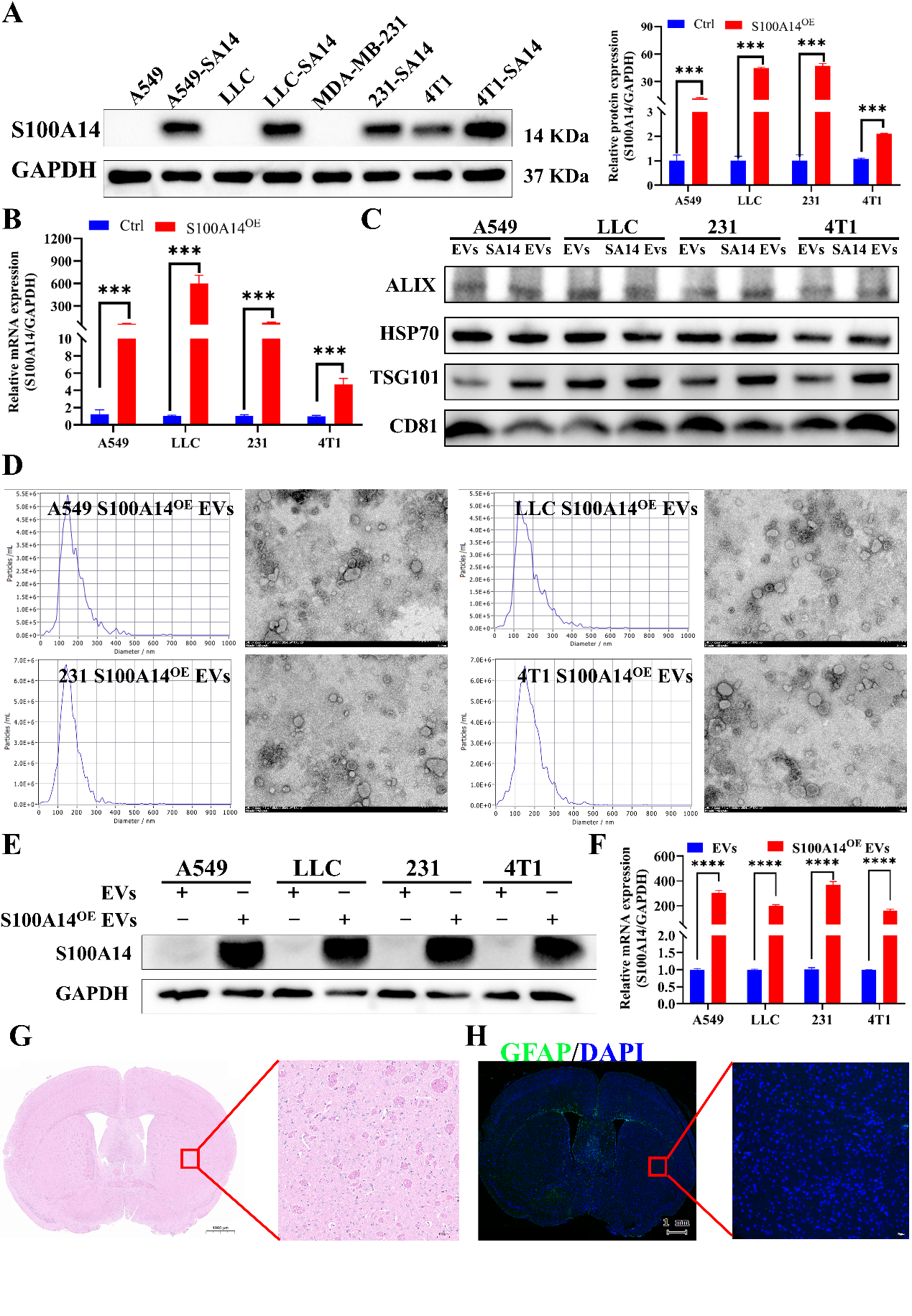


**Figure S1:** Established overexpressed S100A14 cancer cells and extracted S100A14^OE^ EVs. (A) Protein expression levels (left) and quantification (right) of S100A14 in A549-S100A14^OE^ cells, LLC-S100A14^OE^ cells, MDA-MB-231-S100A14^OE^ cells, 4T1-S100A14^OE^ cells and the corresponding parental cells. (B) Quantification of S100A14 mRNA expression levels in A549-S100A14^OE^ cells, LLC-S100A14^OE^ cells, MDA-MB-231-S100A14^OE^ cells, 4T1-S100A14^OE^ cells and the corresponding parental cells. (C) Protein expressions of exosomal markers ALIX, CD81, TSG101 and HSP70 in EVs isolated from A549-S100A14^OE^ cells, LLC-S100A14^OE^ cells, MDA-MB-231-S100A14^OE^ cells, 4T1-S100A14^OE^ cells and the corresponding parental cells. (D) NTA and TEM analysis of EVs isolated from A549-S100A14^OE^ cells, LLC-S100A14^OE^ cells, MDA-MB-231-S100A14^OE^ cells, 4T1-S100A14^OE^ cells. (E) Protein expression levels of S100A14 in A549-S100A14^OE^ EVs, LLC-S100A14^OE^ EVs, MDA-MB-231-S100A14^OE^ EVs, 4T1-S100A14^OE^ EVs. (F) Quantification of S100A14 mRNA expression levels in A549-S100A14^OE^ EVs, LLC-S100A14^OE^ EVs, MDA-MB-231-S100A14^OE^ EVs, 4T1-S100A14^OE^ EVs. (G) The HE staining result of normal brain. (H) IF staining for GFAP (green) and nucleus (blue) in normal brain. Data represent mean ± SEM. Significant difference versus control group, **P*<0.05, ***P*<0.01, and ****P*<0.001.

**Figure S2**


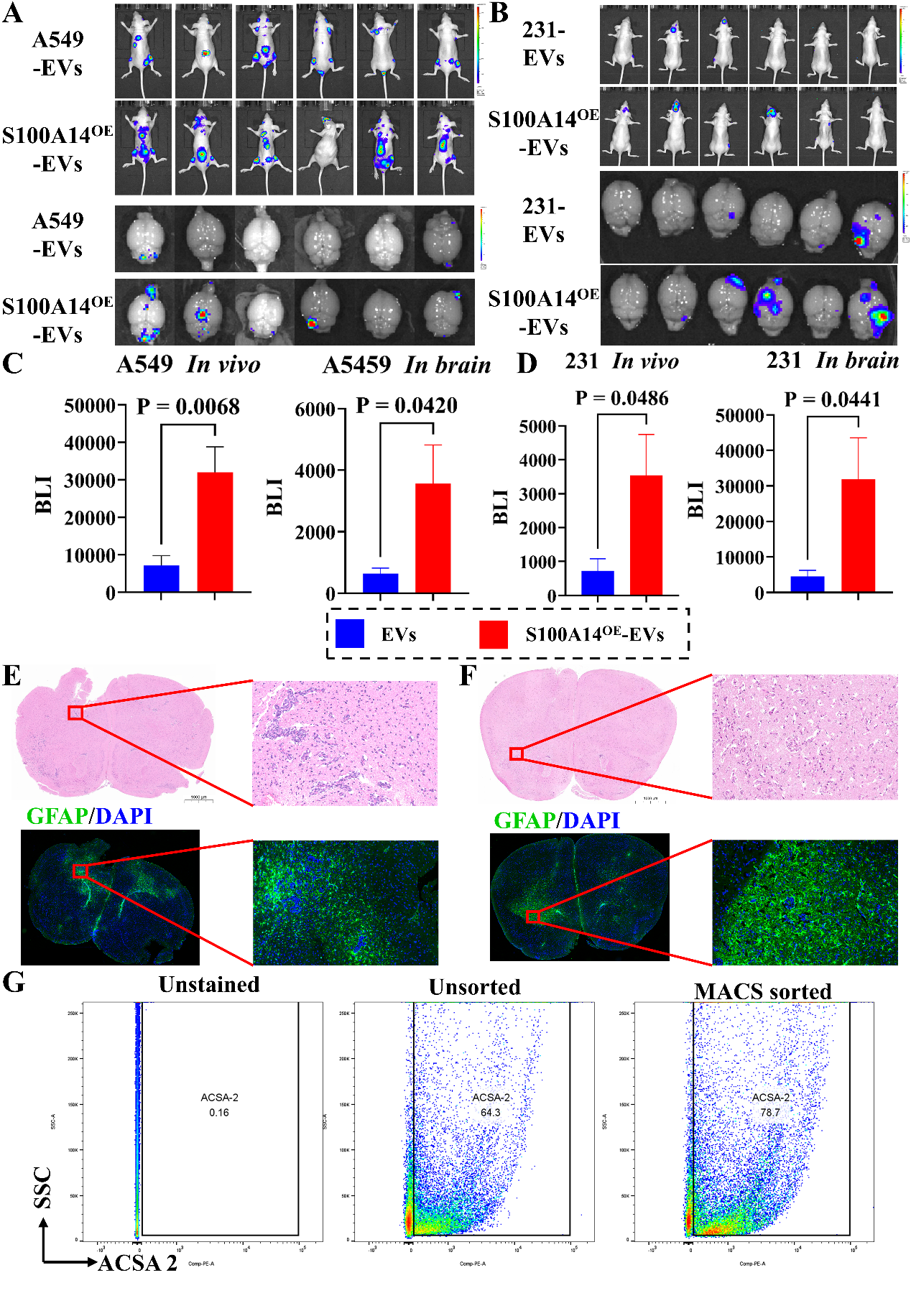


**Figure S2:** *In vivo* and *in brain* bioluminescence imaging (A) and quantification (C) of brain metastatic burden in mice bearing A549 tumors following S00A14^OE^ EVs. *In vivo* and *in brain* bioluminescence imaging (B) and quantification (D) of brain metastatic burden in mice bearing MDA-MB-231 tumors following S00A14^OE^ EVs. (E) The HE staining and GFAP immunofluorescence staining of brain tissue in mice bearing A549 tumors following S00A14^OE^ EVs. (F) The HE staining and GFAP immunofluorescence staining of brain tissue in mice bearing MDA-MB-231 tumors following S00A14^OE^ EVs. (G) Flow cytometry analysis of AS between unsorted and MACS sorted groups. Data represent mean ± SEM. Significant difference versus control group, **P*<0.05, ***P*<0.01, and ****P*<0.001.

**Figure S3**


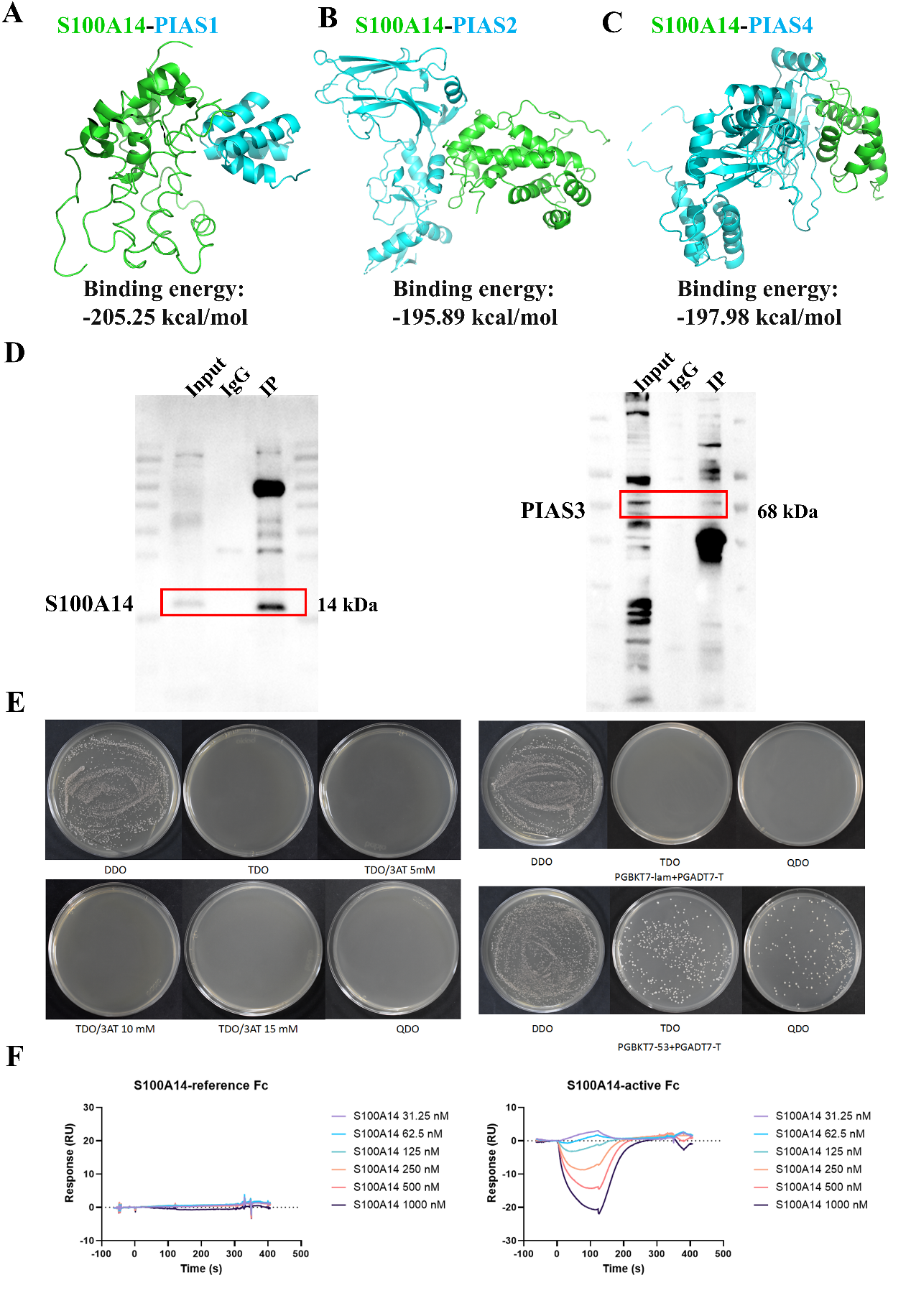


**Figure S3:** The three-dimensional diagrams of S100A14 with PIAS1 (A), PIAS2 (B), and PIAS4 (C) molecular docking results. (D) CO-IP results of S100A14 and PIAS3. (E) Results of Y2H assays of S100A14 self-activation (left) and positive and negative control results of S100a14-PGBKT7 + PIAS3-PGADT7 co-transformation (right). (F) SPR analysis of binding affinity in reference channel.

**Figure S4**


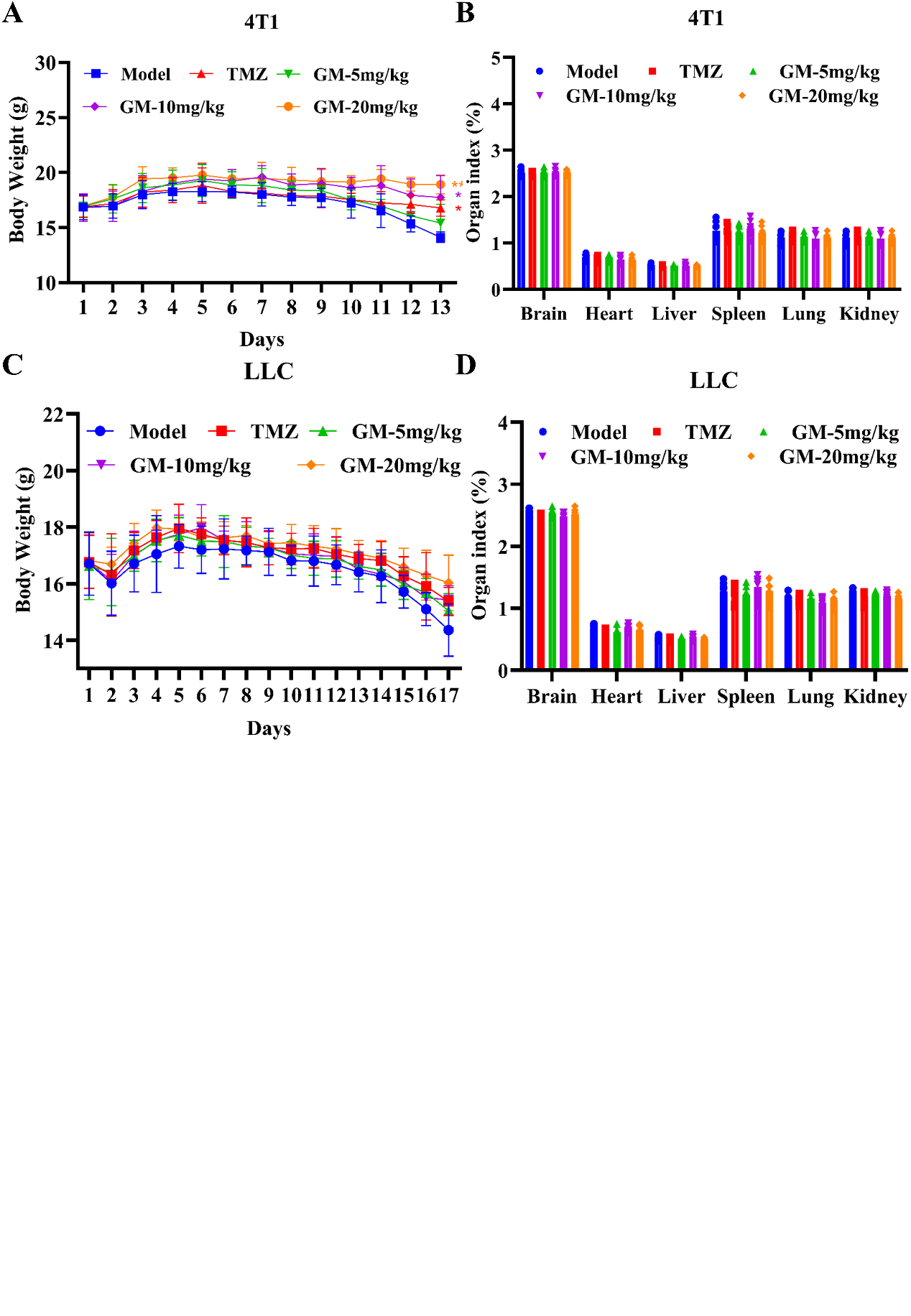


**Figure S4:** The toxicity effects of GM in mice. (A) Body weight of mice bearing 4T1 tumors following S00A14^OE^ EVs in model, TMZ and different concentrations of GM treatment groups. n=9 in each group. (B) Organ index of mice bearing 4T1 tumors following S00A14^OE^ EVs in model, TMZ and different concentrations of GM treatment groups. (C) Body weight of mice bearing LLC tumors following S00A14^OE^ EVs in model, TMZ and different concentrations of GM treatment groups. n=8 in each group. (D) Organ index of mice bearing LLC tumors following S00A14^OE^ EVs in model, TMZ and different concentrations of GM treatment groups. Data represent mean ± SEM. Significant difference versus control group, **P*<0.05, ***P*<0.01, and ****P*<0.001.
